# BB0259 Encompasses a Peptidoglycan Lytic Enzyme Function for Proper Assembly of Periplasmic Flagella in *Borrelia burgdorferi*

**DOI:** 10.3389/fmicb.2021.692707

**Published:** 2021-10-01

**Authors:** Hui Xu, Bo Hu, David A. Flesher, Jun Liu, Md A. Motaleb

**Affiliations:** ^1^Department of Microbiology and Immunology, Brody School of Medicine, East Carolina University, Greenville, NC, United States; ^2^Department of Microbiology and Molecular Genetics, McGovern Medical School, The University of Texas Health Science Center at Houston, Houston, TX, United States; ^3^Department of Microbial Pathogenesis, Yale University School of Medicine, New Haven, CT, United States; ^4^Department of Molecular Biophysics and Biochemistry, Yale University, New Haven, CT, United States; ^5^Microbial Sciences Institute, Yale University, West Haven, CT, United States

**Keywords:** Lyme disease, flagella, peptidoglycan hydrolases, cryo-elecron tomography, spirochete, *Borrelia*

## Abstract

Assembly of the bacterial flagellar rod, hook, and filament requires penetration through the peptidoglycan (PG) sacculus and outer membrane. In most β- and γ-proteobacteria, the protein FlgJ has two functional domains that enable PG hydrolyzing activity to create pores, facilitating proper assembly of the flagellar rod. However, two distinct proteins performing the same functions as the dual-domain FlgJ are proposed in δ- and ε-proteobacteria as well as spirochetes. The Lyme disease spirochete *Borrelia burgdorferi* genome possesses a FlgJ and a PG lytic SLT enzyme protein homolog (BB0259). FlgJ in *B. burgdorferi* is crucial for flagellar hook and filament assembly but not for the proper rod assembly reported in other bacteria. However, BB0259 has never been characterized. Here, we use cryo-electron tomography to visualize periplasmic flagella in different *bb0259* mutant strains and provide evidence that the E580 residue of BB0259 is essential for PG-hydrolyzing activity. Without the enzyme activity, the flagellar hook fails to penetrate through the pores in the cell wall to complete assembly of an intact periplasmic flagellum. Given that FlgJ and BB0259 interact with each other, they likely coordinate the penetration through the PG sacculus and assembly of a functional flagellum in *B. burgdorferi* and other spirochetes. Because of its role, we renamed BB0259 as flagellar-specific lytic transglycosylase or LTase^Bb^.

## Introduction

In most bacterial species, the flagellum is a rotary nanomachine framed in a supramolecular structure that initiates assembly from the cell envelope and extends into the extracellular space. However, in motile spirochetes, this migratory organelle resides in the periplasmic space and is therefore called the periplasmic flagellum (Wolgemuth et al., 2006; Charon et al., 2009, 2012). Despite differences in location, the external flagella found in the model organisms *Escherichia coli* and *Salmonella enterica* serovar Typhimurium share a remarkably high degree of similarity with respect to overall structure, as the key components are conserved among the external flagella and the periplasmic flagella of spirochetes (Liu et al., 2009; Chen et al., 2011; Zhao et al., 2013; Terashima et al., 2017; Carroll and Liu, 2020). The supramolecular structure is composed of more than 25 distinct protein subunits and self-assembles to final lengths of up to 20μm in the model organisms. The individual flagellum consists of three major substructures: a basal body, hook, and filament (reviewed in Nakamura and Minamino, 2019; Carroll and Liu, 2020). Synthesis of the flagellum begins with the assembly of a basal body composed of an MS ring embedded in the cell membrane and a rod. The MS ring is comprised of multiple copies of FliF and acts as a platform for assembly of the rod, C-ring, torque-generating stator, and flagellum-specific type III secretion system (fT3SS). The rod is composed of five proteins, FliE, FlgB, FlgC, FlhO/FlgF, and FlgG. These rod proteins are transported from the cytoplasm to the periplasmic space *via* the fT3SS (Saijo-Hamano et al., 2019) and then assemble onto the MS ring—in an ordered fashion from the proximal rod to the distal rod (Zhao et al., 2013; Terashima et al., 2017; Carroll and Liu, 2020). The distal rod serves as the template for subsequent assembly of the P-ring and hook (*B. burgdorferi* lacks the L-ring; Zhao et al., 2013). The filaments are subsequently polymerized onto the hook to complete assembly of a flagellum. Hook and filament assembly is mediated by the hook cap FlgD and filament cap FliD protein, respectively (Homma and Iino, 1985; Ohnishi et al., 1994; Yonekura et al., 2000).

In order for the assembly of the rod with 30nm×4nm to penetrate the outer membrane in *E. coli* or *S. enterica*, a hole must be created in the peptidoglycan sacculus, as its mesh diameter is narrower than 2nm (Demchick and Koch, 1996; Herlihey et al., 2014). This PG hydrolytic activity is accomplished by a dual-domain protein FlgJ of *S. enterica* and *E. coli*. FlgJ is composed of an N-terminal scaffolding domain required for polymerization of the distal rod and a C-terminal domain required to form a hole in the PG sacculus. The C-terminal domain of FlgJ proteins belongs to the glycoside hydrolase family in which these β-N-acetyl-glucosaminidases cleave the β-1,4 glycosidic linkage between the N-acetylglucosamine and N-acetylmuramic acid residues of bacterial cell wall peptidoglycan, with Glu184 and Glu223 serving as the catalytic residues (Nambu et al., 1999; Herlihey et al., 2014; Zaloba et al., 2016).

Once the enzyme has made a hole in the cell wall, FlgG proteins are secreted and assemble in two stacks to make an 11nm-long rod just underneath the FlgJ rod cap, thereby spanning the remaining distance between the cell wall and outer membrane (Jones and Macnab, 1990; Cohen and Hughes, 2014). The ejection of the FlgJ rod cap allows the FlgD hook cap to assemble at the tip of the rod and results in the transition from rod to hook polymerization (Nambu et al., 1999; Cohen and Hughes, 2014). While dual-domain FlgJ proteins are found in most β- and γ-proteobacteria, two distinct proteins performing similar functions as the dual-domain FlgJ are reported in diverse bacterial genomes, including δ- and ε-proteobacteria as well as spirochete species *B. burgdorferi*, *Leptospira interrogans*, and *Treponema* (Nambu et al., 1999, 2006; Gonzalez-Pedrajo et al., 2002; Zaloba et al., 2016; Garcia-Ramos et al., 2018). Moreover, the single-domain flagellar scaffolding protein FlgJ is reported to interact with PG-lytic enzymes, such as soluble lytic transglycosylases (SLT) proteins (Garcia-Ramos et al., 2018). These specialized cell wall-degrading enzymes allow the efficient assembly and anchoring of supramolecular structures in the cell envelope. The SLTs represent one class of autolysins that act like a lysozyme (muramidase) to cleave the β-(1→4) glycosidic linkage between N-acetylmuramyl and N-acetylglucosaminyl residues of cell wall peptidoglycan. Unlike lysozymes, however, SLTs are not hydrolases but cleave the β-glycosidic linkages, with simultaneous production of 1,6-anhydromuramyl residues (Holtje et al., 1975; van Asselt et al., 1999; Blackburn and Clarke, 2001; Koraimann, 2003).

As *B. burgdorferi* FlgJ is reported to be a single-domain protein that lacks a PG-hydrolyzing enzymatic domain, we sought to investigate the genome to identify a protein that harbors this domain (Zhang et al., 2012). BB0531 and BB0259 identified in this communication are both predicted to possess PG-hydrolyzing enzyme activities. BB0531 possesses a glucosaminidase domain; however, subsequent analysis indicates that this protein is not involved in flagellar pore-forming activities. BB0259 possesses an SLT homolog at its C-terminal domain, and further analyses indicate that FlgJ specifically interacts with BB0259. Because of its apparent flagellar specific peptidoglycan lytic enzyme function in the spirochete, BB0259 is renamed as LTase^Bb^. FlgJ and LTase^Bb^ appear to function distinctively compared to their counterparts from other bacteria. Our model describes how *B. burgdorferi* FlgJ and LTase^Bb^ work synchronously to form a functional periplasmic flagellum.

## Materials and Methods

### Bacterial Strains and Growth Conditions

*Borrelia burgdorferi* B31-A is a high-passage noninfectious clone used as the wild-type (WT) strain (Bono et al., 2000; Tilly et al., 2001). The isogenic *bb0259* mutant and complemented strains were constructed as described below. All *B. burgdorferi* strains were grown in Barbour–Stoenner–Kelly II (BSK-II) liquid medium or on soft agarose plates at 35°C in the presence of 2.5% CO_2_ (Motaleb et al., 2007). Antibiotics, when needed, were supplied in BSK-II medium at proper concentrations: 200μg/ml kanamycin and/or 40μg/ml gentamycin. All *Escherichia coli* strains were cultured in Luria-Bertani (LB) liquid medium or on LB agar plates (Bertani, 1951, 2004). When required, 100μg/ml ampicillin or 4μg/ml gentamycin was supplemented into the LB medium.

### Bioinformatics

Basic local alignment search tool (BLAST) was used to determine protein homologs from the sequence database (Altschul et al., 1990, 1997). For the BLAST analysis, a significant homolog is determined based on their E-value [the lower an E-value (the closer it is to zero), the more significant the score is]. Signal peptide was predicted using SignalP 5.0 (Almagro Armenteros et al., 2019) and Phobius (Kall et al., 2004, 2005, 2007). Analysis of bacterial lipoprotein was performed using DOLOP (Madan Babu and Sankaran, 2002; Babu et al., 2006). Clustal Omega was utilized to align multiple amino acid sequences (Sievers et al., 2011). FFAS server was used as described elsewhere.

### Overexpression and Purification of Recombinant Proteins in *Escherichia coli*

Multiple attempts to express soluble *B. burgdorferi* BB0259 with variable lengths using short-affinity tags (His6 or Strep) failed, as all these recombinant BB0259 proteins with short affinity tags formed inclusion bodies in the *E. coli* host. In order to produce soluble recombinant BB0259 protein, a DNA fragment harboring the BB0259 open reading frame (ORF) without signal peptide (1-49 aa) was polymerase chain reaction (PCR)-amplified from chromosomal DNA of *B. burgdorferi* B31-A using the primers PF rBB0259-BamHI (GGATCC TGGTTATGGAATTTTGATTATAC) and PR rBB0259-PstI (CTGCAGTTAATTTTTGGGGAATTCGCCC; restriction sites are underlined) and cloned into the pMAL c5x (NEB Inc.) through *Bam*HI and *Pst*I restriction sites to produce maltose binding protein (MBP)-tagged BB0259. Similarly, the 1xFLAG (DYKDDDDK) tagged FlgJ with gene locus number (BB0771a, also named BB0858) was constructed for far-western or affinity blotting. A DNA fragment of full-length *flgJ* fused with 1xFLAG tag coding sequence (GACTACAAAGACGATGACGACAAG) at C-terminus was amplified by PCR with primers PF MBPFlgJBamHI (CGTCGACGGATCCGAAACCAAAATTAAT TCACAAAATC) and PR MBPFlgJPstI (TAATTACCTGCAG TTACTTGTCGTCATCGTCTTTGTAGTCTTTACTTTTTTGTA ATTGATTGTA) and cloned into the pMAL c5x using *Bam*HI and *Pst*I restriction sites. MBP tagged FlaB (BB0147), FlgE (BB0283), FlgD (BB0284), FlgG (BB0774), and FlgL (BB0182) were also prepared to determine protein–protein interactions. The coding regions of FlaB, FlgE, FlgG, and FlgL were amplified by PCR with primers PF MBPflaB_NotI (GTCCATGGGCGG CCGCATGATTATCAATCATAATACATC) and PR MBPflaB_BamHI (GGAATTCGGATCCTTATCTAAGCAATGACAAAA CATATTG), PF MBPflgE_NotI (GTCCATGGGCGGCCGC ATGAT GAGGTCTTTATATTCTGG) and PR MBPflgE_BamHI (GGAATTCGGATCCTTAATTTTTCAATCTTACAAGTTCTTG), PF MBPflgG_NotI (GTCCA TGGGCG GCCGCATGATGAGA GCATTATGGACAGC) and PR MBPflgG_BamHI (GGAATTC GGATCC TTATTGCCTTTTTAAGTTATTTGC), PF MBPflgL_NotI (GTCCATGGGCGGCCGCATGATAAATAGAGTAAGTCA TCC) and PR MBPflgL_BamHI (GGAATTCGGATCCTTATTT TATAAAATCTAATAAAGTCG), respectively, and cloned into the pMAL c5x (NEB Inc.) using *Not*I and *Bam*HI restriction sites. Recombinant FlgD construct was similarly prepared using *Nde*I-*Nco*I restriction enzymes with primers PF MBPflgD_NdeI (GCTGGACGACATATGAATAAAATAAACGGTGTTGAAAATG) and PR MBPflgD_NcoI (TGCTGGGCACCATGG TTATTCC TCCAAACCTACCGATAAT). All *E. coli* DH5α strains carrying the pMAL c5x constructs for expressing BB0259, FlaB, FlgE, FlgG, FlgL, FlgD, 1xFLAG tagged FlgJ were induced with 0.25mM Isopropyl β-D-1-thiogalactopyranoside (IPTG) at room temperature, and purifications of recombinant proteins were performed with amylose resin (NEB Inc.) according to the manufacturer’s protocol.

### SDS-PAGE, Immunoblot, and Affinity Blotting

Sodium dodecyl sulfate–polyacrylamide gel electrophoresis (SDS-PAGE) was carried out as mentioned previously (Motaleb et al., 2000). Exponentially growing *B. burgdorferi* cells were harvested and washed with phosphate-buffered saline (PBS) and resuspended in the same buffer to process the preparation of cell lysate for SDS-PAGE. Immunoblotting was performed with *B. burgdorferi* FlaB, FlgE, MotB, FliL, and DnaK-specific antibodies (Motaleb et al., 2000, 2011; Sal et al., 2008; Sultan et al., 2015) and Pierce^™^ ECL or ECL 2 substrate (Thermo Fisher Scientific Inc.). Concentration of the proteins was determined using a Bio-Rad protein assay kit with bovine serum albumin (BSA) as the standard. Unless specified, approximately 10μg of cell lysates was subjected to SDS-PAGE.

Far-western or affinity blotting assay with recombinant proteins was performed as described previously (Toker and Macnab, 1997; Kariu et al., 2015; Moon et al., 2016, 2018; Xu et al., 2019). Briefly, 1μg purified recombinant proteins was subjected to SDS-PAGE and Coomassie blue staining or transferred to polyvinylidene difluoride (PVDF) membrane. The PVDF membranes were blocked in the blocking solution (5% BSA, 10mM Tris, 150mM NaCl and 0.3% Tween 20, pH 7.4) with gentle shaking for 4 to 6h at room temperature, and then the membranes were incubated with or without the purified FLAG-FlgJ protein at the concentration 2μg/ml in blocking solution overnight. The membranes were washed 3 times with the washing buffer (10mM Tris, 150mM NaCl and 0.3% Tween 20, pH 7.4) and then immunoblotted with monoclonal anti-FLAG^®^ M2 antibody (Sigma-Aldrich Co. LLC) followed by ECL 2 detection, as mentioned above. The X-ray films were exposed to the membranes for less than a minute.

### Construction of the *Borrelia burgdorferi* Mutants and Complemented Strains

Construction of the *bb0259* and *bb0531* inactivation plasmids, electroporation, and plating of *B. burgdorferi* were as described earlier (Motaleb et al., 2000; Sultan et al., 2010, 2013; Moon et al., 2016; Xu et al., 2017). *bb0259* was inactivated by *P_flgB_*-kanamycin resistance cassette (*P_flgB_-aph1*), which possessed the *flgB* promoter driving kanamycin resistance gene (Ge et al., 1997; Bono et al., 2000; Li et al., 2010; Zhang et al., 2012; Moon et al., 2016; Xu et al., 2017, 2019). In short, DNA fragment including upstream 394-bp region of *bb0259*, *bb0259* containing five *Hind*III sites, and downstream 324-bp region of *bb0259* was amplified by PCR from chromosomal DNA of wild-type *B. burgdorferi* strain B31-A through Expand^™^ High Fidelity PCR System (Sigma-Aldrich Inc.) using primers BB0259-KO-F (GTTGAGTATATTGACGAGAAG) and BB0259-KO-R (TCCCAACAA CTCCGGTAACA), which was further TA cloned into pGEM-T Easy (Promega Inc.), yielding pGEM-T Easy::*bb0259*. *P_flgB_-Kan* was introduced into pGEM-T Easy::*bb0259* through the first and last *HindIII* sites located within *bb0259* gene, creating pGEM-T Easy::*bb0259KO-P_flgB_-Kan*, of which the direction of transcription of *P_flgB_-Kan* was confirmed to be the same as that of *bb0259*. Plasmid pGEM-T Easy::*bb0259KO-P_flgB_-Kan* was linearized by *Not*I restriction digestion and electroporated into competent *B. burgdorferi* B31-A cells to create ∆*bb0259* mutant cells. *bb0531* mutant was similarly prepared using a promoter-less kanamycin cassette (*Pl-Kan*), as described (Sultan et al., 2010). Kanamycin-resistant transformants were screened by PCR to confirm the genotypes of the mutants.

The *bb0259* mutant was complemented *in trans* using the *B. burgdorferi*-*E. coli* shuttle vector pBSV2G (Elias et al., 2003). In short, *bb0259* was PCR amplified using primers BB0259 Comp F (GATCCATATGTTTAATAGAAGTTCTTG; underlined sequence is *NdeI* site) and BB0259 Comp R (CTGCAGTTAATTT TTGGGGAATTCGCCC) and ligated into pGEM-T Easy, yielding plasmid *bb0259*ORF-Easy. DNA fragment containing *bb0259* ORF was then released using *NdeI* restriction digestion from *bb0259*ORF-Easy, and further ligated into *NdeI*-treated P_flgB_-motB-Easy vector (Sultan et al., 2015), creating plasmid P_flgB_-bb0259-Easy. Finally, this plasmid was digested with *NotI* and cloned into the same restriction site of the shuttle vector pBSV2G, generating pBSV2G::*P*_flgB_-*bb0259*. The newly constructed shuttle vector pBSV2G::*P*_flgB_-*bb0259* was electroporated into the ∆*bb0259* mutant cells, followed by selection with both kanamycin and gentamycin. The resistant clones, namely *bb0259^com^*, were analyzed by PCR for confirmation of the presence of plasmid pBSV2G::*P*_flgB_-*bb0259*.

*Borrelia burgdorferi bb0259-*E580Q (E glutamate to Q glutamine) and *bb0259-*D606N (D aspartate to N asparagine) point-mutated strains were constructed through allelic exchange mutagenesis (Yang et al., 2003). Simply, using B31-A chromosomal DNA as the template, the left arm (882bp, partial *bb0259* gene), promoter-less kanamycin cassette (*Pl-Kan*; 846bp), and right arm (564bp, including 88bp leftover from *bb0259* gene for potential ribosome binding and 476bp of partial *bb0258* gene) were PCR amplified, respectively, using primer pairs P1F BB0259PointMuKOplKan (CCTAACGTAAGCGGAGAATACA AGAGTCTTTTGCATTCTG) and P1R BB0259Point MuKO plKan (TAAAATTGCTTTTAACTATTAATTTTTGGGGAATTC GCCC), P2F BB0259PointMuKOplKan (GGGCGAATTCCCC AAAAATTAATAGTTAAAAGCAATTTTA) and P2R BB0259 PointMuKOplKan (CTTTTCATACAAAGCATCATTTAGAAA AACTCATCGAGC), P3F BB0259PointMuKOplKan (GCTCGA TGAGTTTTTCTAAATGATGCTTTGTATGAAAAG) and P3R BB0259PointMuKOplKan (CCCAAGCCTTGCATCAGCCCCA TAAAAATTCCTGCTAAC). Through overlapping PCR, *Pl-Kan* was inserted between the left arm and right arm, and further cloned into pGEM-T Easy, creating vector pGEM-T Easy:: bb0259PointMuKO-Pl-Kan. Using pGEM-T Easy::bb0259 PointMuKO-Pl-Kan as the template, point mutations of BB0 259E580Q were introduced by QuikChange II Site-Directed Mutagenesis Kit (Agilent Technologies Inc.) using the primers PF BB0259E580Q (CTTTAAT AAAAGCACAA AGTAG CTTTG AAAAAAATG) and PR BB0259E580Q (CATTTTTTTCAA AGCTACTTTGT GCTTTTATTAAAG; bold and underlined sequences indicate the point mutation), to create pGEM-T Easy::bb0259E580QKO-Pl-Kan. Similarly, through site-directed mutagenesis pGEM-T Easy::bb0259D606NKO-Pl-Kan was generated with primers PF BB0259D606N (GCCATCAACAGC AAATAATATTTCTAAA GAACTTAAG) and PR BB0259D606N (CTTAAGTTCTTTA GAAATATTATTTGC TGTTGATGGC). These two vectors were linearized by *Not*I digestion and electroporated into the competent *B. burgdorferi* B31 A cells to generate *bb0259-*E590Q and *bb0259-*D606N mutant strains, of which the point mutations of *bb0259* were further verified by sequencing.

### Dark-Field Microscopy and Swarm Plate Motility Assays

Exponentially growing *B. burgdorferi* cells were observed using a Zeiss Axio Imager M1 dark-field microscope to determine bacterial morphology and motility. To evaluate the motility of *B. burgdorferi* cells, swarm plate assays were performed following our well-established protocol (Motaleb et al., 2000, 2007, 2011).

### Cryo-ET Analysis of *bb0259* and *bb0531* Mutant Cells

Frozen-hydrated *B. burgdorferi* specimens were prepared as described previously (Zhao et al., 2013). In short, the bacterial culture was centrifuged at 5,000×*g* for 5min, and the resulting pellets were suspended in PBS to achieve a cell concentration ~1×10^8^/ml. After adding 10-nm gold marker solution, 5μl of the cell suspension was placed on freshly glow-discharged holey carbon grid (Quantifoil Cu R2/2, 200 mesh) for 25s. The grids were blotted with filter paper for 3 to 5s and rapidly frozen in liquid ethane, using a homemade plunger apparatus as described previously (Zhao et al., 2013).

The Δ*bb0531*, Δ*bb0259*, and complemented *bb0259^com^* strains were imaged at −170°C using a Polara G2 electron microscope (FEI Company) equipped with a field emission gun and a 4,096-by-4,096 charge-coupled device (16-megapixel) camera (TVIPS Gmbh, Germany). The microscope was operated at 300kV with a magnification of ×31,000, resulting in an effective pixel size of 5.7Å. Using the FEI batch tomography program, low-dose single-axis tilt series were collected from each bacterium at ~8μm defocus with a cumulative dose of ~100 e^−^/Å^2^ distributed over 65 images. In total, 80 tomographic reconstructions of Δ*bb0259* cell tips and 10 tomographic reconstructions of complemented *bb0259* cells were generated.

The *bb0259* point mutants (*bb0259*-E580Q and *bb0259*-D606N) were imaged with Krios electron microscope (Thermo Fisher) with a field emission gun, a volta phase plate, and a direct electron detector (Gatan K3 Summit). SerialEM was used to collect tilt series at focus. A total dose of 55 e^−^/Å^2^ is distributed among 35 tilt images covering angles from −51° to +51° at tilt steps of 3°. For every single tilt series collection, dose-fractionated mode was used to generate 11 frames per projection image. Collected dose-fractionated data were first subjected to the MotionCor2 to generate drift-corrected files (Zheng et al., 2017). IMOD software was used to align the tilt series and to generate tomograms (Kremer et al., 1996). In total, 240 tomographic reconstructions of *bb0259*-E580Q cells and 10 tomographic reconstructions of *bb0259*-D606N cells were generated.

### Subtomogram Averaging

A total of 646 flagellar motor subtomograms were picked from Δ*bb0259* tomograms. The initial orientation of each particle was manually estimated by the C-ring and hook. The subtomograms were used to generate the averaged structure as described previously (Zhao et al., 2013).

### Three-Dimensional Visualization

The software package EMAN2 was used for 3D segmentation (Chen et al., 2017). Segmentation of tomograms was performed using supervised machine learning to segment features of interest. The outer membrane, inner membrane, peptidoglycan layer, and flagella were segmented in this manner and manually cleaned using ChimeraX (Goddard et al., 2018). An existing motor model derived from subtomogram averaging was manually inserted into the segmented model. ChimeraX was used for visualization.

## Results

### An Open Reading Frame Encompasses a Peptidoglycan-Hydrolyzing Enzymatic Domain

The single-domain FlgJ of *B. burgdorferi* plays roles in hook and filament assembly but not in rod assembly in *S. enterica* (Nambu et al., 1999; Cohen and Hughes, 2014; Zaloba et al., 2016), as *flgJ* mutant cells possess an intact rod and P-ring but form partial hook and filament structures (Zhang et al., 2012). Given that an enzymatic activity is required for pore formation in the PG layer for assembly of the flagellar hook and filament, and that *B. burgdorferi* FlgJ lacks a peptidoglycan-hydrolyzing enzyme domain, we sought to determine an ORF containing an enzymatic domain in the Lyme disease spirochetal genome. Using the *S. enterica* FlgJ with GenBank accession number NP_460153 as a query in BLAST search, we found no significant homologs in *B. burgdorferi* (not shown; Nambu et al., 1999; Hirano et al., 2001; Cohen and Hughes, 2014). However, using the FFAS server,^1^ BB0531 was identified as the only significant homolog of FlgJ (homology score −41.70; a lower FFAS score indicates higher confidence of the prediction; Rychlewski et al., 2000; Jaroszewski et al., 2005, 2011). BB0531 is annotated as a hypothetical protein with unknown function in the *B. burgdorferi* genome; however, it is predicted to possess a mannosyl-glycoprotein endo-β-N-acetylglucosaminidase domain responsible for peptidoglycan remodeling or hydrolyzing activity (Pfam: PF01832). BB0531 shares only 15% amino acid sequence identity with *S. enterica* FlgJ and alignment of amino acid sequences of *S. enterica* FlgJ and BB0531 from FFAS shows that the catalytic residues of FlgJ are not well conserved in BB0531 leading us to speculate that BB0531 is not involved in peptidoglycan hydrolyzing activity for flagellar penetration (not shown).

**Figure 1:**
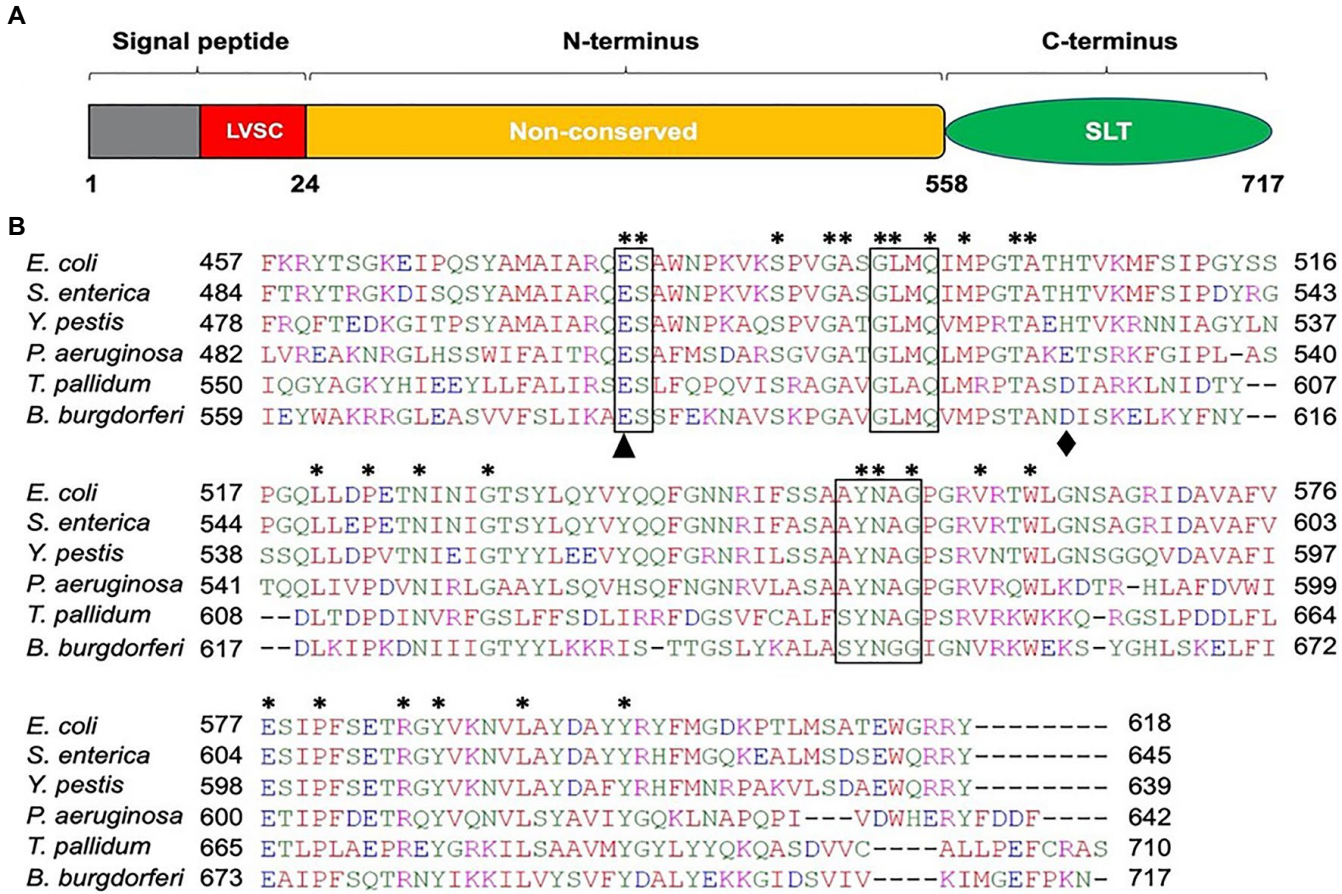
Amino acid sequence analysis of *B. burgdorferi* BB0259 (GenBank accession no. NP_212393). **(A)** Domain architecture of BB0259. BB0259 consists of 717 amino acids and is predicted to contain a lipobox consensus sequence (LVSC) in the signal peptide region (1-24 aa) determined by DOLOP program: https://www.mrc-lmb.cam.ac.uk/genomes/dolop/analysis.shtml. BB0259 also possesses a non-conserved N-terminal domain (25-558 aa) and a conserved C-terminal soluble lytic transglycosylase (SLT) domain (559-717 aa). Diagram is not in scale. **(B)** Multiple sequence alignment of SLT domains with GenBank accession no. 1QSA_A from *E. coli*, APW71272 from *S. enterica*, ANW15826 from *Y. pestis*, AID74248 from *P. aeruginosa*, and WP_010881492 from *Treponema pallidum* using Clustal Omega program: https://www.ebi.ac.uk/Tools/msa/clustalo/. Asterisks indicate identical residues. Boxed regions denote the conserved motifs ES-GLMQ-AYNAG among the SLT-domain proteins. *S. enterica* SLT domain shares 35% identity with the SLT domain of BB0259 with E-value 6e−25. Black triangle and diamond demonstrate the catalytic (E580) and non-conserved (D606) residues, respectively.

Because *B. burgdorferi* lacks a significant homolog of *S. enterica* FlgJ, we used the model organism’s SLT domain as a query in the BLAST search and identified BB0259 as a homolog in *B. burgdorferi*. BB0259 shares 35–41% amino acid sequence identity with significant E-value at the SLT domain (Figure 1; Pfam: PF01464; Thunnissen et al., 1994; van Asselt et al., 1999; Koraimann, 2003). Importantly, the motifs and amino acid residues required for peptidoglycan lytic enzymatic activity are highly conserved in BB0259 (Figure 1). BB0259 possesses a signal sequence (1-24 aa) with lipobox consensus residues LVSC, an unknown domain with no homologs (25-558 aa), and an SLT domain at the C-terminal region (559-717 aa; Figure 1). The presence of these features led us to hypothesize that BB0259 is secreted into the periplasmic space using its signal sequence to create pores in the PG layer, thereby completing assembly of the flagellar hook–filament.

### Mutants in *bb0259*, but Not *bb0531*, Exhibit Defective Motility and Altered Morphology

**Figure 2:**
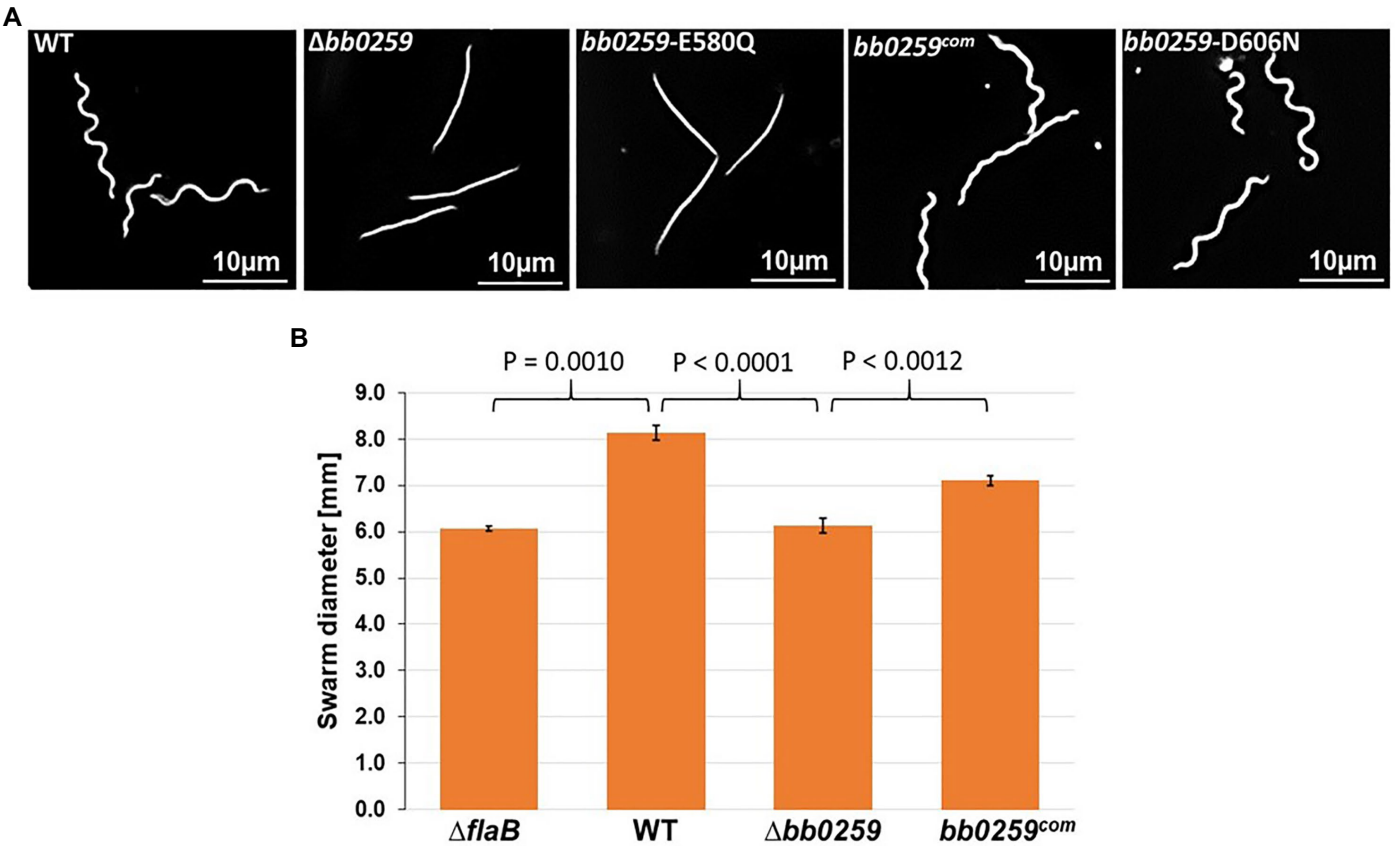
Morphology and motility phenotypes of the *bb0259* mutant cells. **(A)** Dark-field microscopic images showing the characteristic flat-wave morphology of the WT, complemented *bb0259^com^* and *bb0259-*D606N cells and rod-shaped morphology of the Δ*bb0259* and *bb0259-*E580Q mutants. **(B)** Average swarm diameters from three swarm plates are shown in millimeter scale. *bb0259*-E580Q and *bb0259*-D606N mutants show motility phenotypes similar to the Δ*bb0259* and WT cells, respectively, and are not shown. A non-motile flagellar filament mutant Δ*flaB* was used as a control. Bars represent mean±standard deviation of the mean from three plates. Values of *p* between samples are shown at the top. A value of *p*<0.05 was considered as significantly different.

Completion of the appropriately assembled flagellar rod, hook, and filament is critical for the spirochete motility and morphology (Motaleb et al., 2000, 2015; Sal et al., 2008; Sultan et al., 2013; Zhao et al., 2013). If the SLT domain of BB0259 or the glucosaminidase domain of BB0531 displays PG-hydrolyzing activities, which are specific for flagellar assembly, then a deletion mutant in *bb0259* or *bb0531* is expected to be unable to create pores in the PG layer, resulting in defective rod/hook/filament assembly and a motility-deficient phenotype. To test this proposition, we created deletion mutants Δ*bb0259* and Δ*bb0531* (Supplementary Figures S1, S2, and S5). While the Δ*bb0531* cells lack any observable phenotype with respect to motility and morphology, the Δ*bb0259* mutant cells observed under a dark-field microscope are completely non-motile (Figure 2A and not shown). Swarm plate motility assays also indicated that Δ*bb0259* mutant cells are deficient in swarming out from the initial site of inoculation in soft agarose plates (Figure 2B). Furthermore, while wild-type cells show characteristic flat-wave morphology, Δ*bb0259* mutant cells are rod-shaped (Figure 2A). To demonstrate that the phenotypes described here are devoid of a polar effect on downstream gene expression or a secondary mutation elsewhere in the genome, we complemented the mutant *in trans* using a shuttle vector (Supplementary Figures S1, S2). Complemented *bb0259* (*bb0259^com^*) cells observed under a microscope as well as in swarm plate motility assays indicated morphology and motility phenotypes restored to wild-type level (Figures 2A,B). Furthermore, SLT proteins important for hydrolyzing the PG sacculus for flagellar insertion and subsequent assembly of an intact flagellum are reported to be secreted in the periplasm using their Sec-signal peptide (Garcia-Ramos et al., 2018). Consistent with this observation, a recent study reported that BB0259 is associated with the inner membrane (Toledo et al., 2018). To determine whether the Sec-signal sequence is required for BB0259 proteins to be secreted in the periplasm, where they create pores in the PG sacculus for flagellar assembly and thereby provide motility to the cell, we complemented the non-motile Δ*bb0259* mutant without the signal sequence (*bb0259*Δsec). As described above, while the full-length *bb0259* was able to complement the Δ*bb0259* mutant and therefore restored the morphology and motility phenotypes (Figures 2A,B), the Sec-signal sequence-deleted variant *bb0259*Δsec was unable to restore any of the phenotypes (data not shown). These results suggest that the signal sequence is essential for BB0259 proteins to be secreted in the periplasm.

The altered motility and morphology phenotypes exhibited by the deletion mutant suggest that the rod/hook/filament structure was not properly assembled due to a lack of the SLT enzymatic activity of BB0259 that would hydrolyze the PG layer for flagellar structures to assemble through the PG sacculus and provide motility to the bacterial cell. *B. burgdorferi* cells lacking the gene-encoding filament FlaB or hook FlgE were reported to be non-motile and rod-shaped (Motaleb et al., 2000; Sal et al., 2008; Sultan et al., 2013). We therefore tested whether these gene products were synthesized in the Δ*bb0259* cells. Western blotting results (Supplementary Figure S3) indicate that the mutant cells synthesized very few FlaB proteins, with no effect on synthesis of the hook protein FlgE. Lack of the filaments in the Δ*bb0259* cells may thus have resulted in the motility-deficient and rod-shaped morphology (Figures 2A,B).

### Glutamate E580 of BB0259 Is the Catalytic Site Residue

The SLT enzyme domain proteins possess a conserved ES-GLMQ-AYNAG motif in which the glutamate [E] residue is reported to be the catalytic residue (Figure 1; Thunnissen et al., 1994; van Asselt et al., 1999; Koraimann, 2003). The SLT domain identified in the C-terminal region of BB0259 possesses an E residue at position 580. To determine whether the E residue is the catalytic site, we created a point mutant in E580 of BB0259, resulting in *bb0259*-E580Q *B. burgdorferi* (Supplementary Figures S1, S2). Furthermore, the E223 and D248 residues of the *S. enterica* FlgJ enzymatic domain were reported to be important for PG-hydrolyzing activities (Nambu et al., 1999), and we noticed that the BB0259 SLT domain also encompasses similarly distanced E580 and D606 residues (Figure 1). Even though BB0259 shares limited homology with *S. enterica* FlgJ, and E580 and D606 do not align with E223 and D248 residues of FlgJ (not shown), we created a *bb0259*-D606N *B. burgdorferi* point mutant to determine whether this non-conserved D606 residue codes for any enzyme function (Supplementary Figures S1, S2). The *bb0259*-E580Q point mutant *B. burgdorferi* cells exhibited phenotypes identical to those of the deletion mutant Δ*bb0259* with respect to morphology and motility, whereas the *bb0259*-D606N cells were devoid of any detectable phenotype (Figures 2A,B). These results indicate that E580 of BB0259 is the catalytic site residue required for BB0259 function, while the D606 residue is not important for PG-hydrolyzing activities.

### Rod/Hook Structures of *bb0259* Mutant Cells Lie Underneath the PG Layer

**Figure 3:**
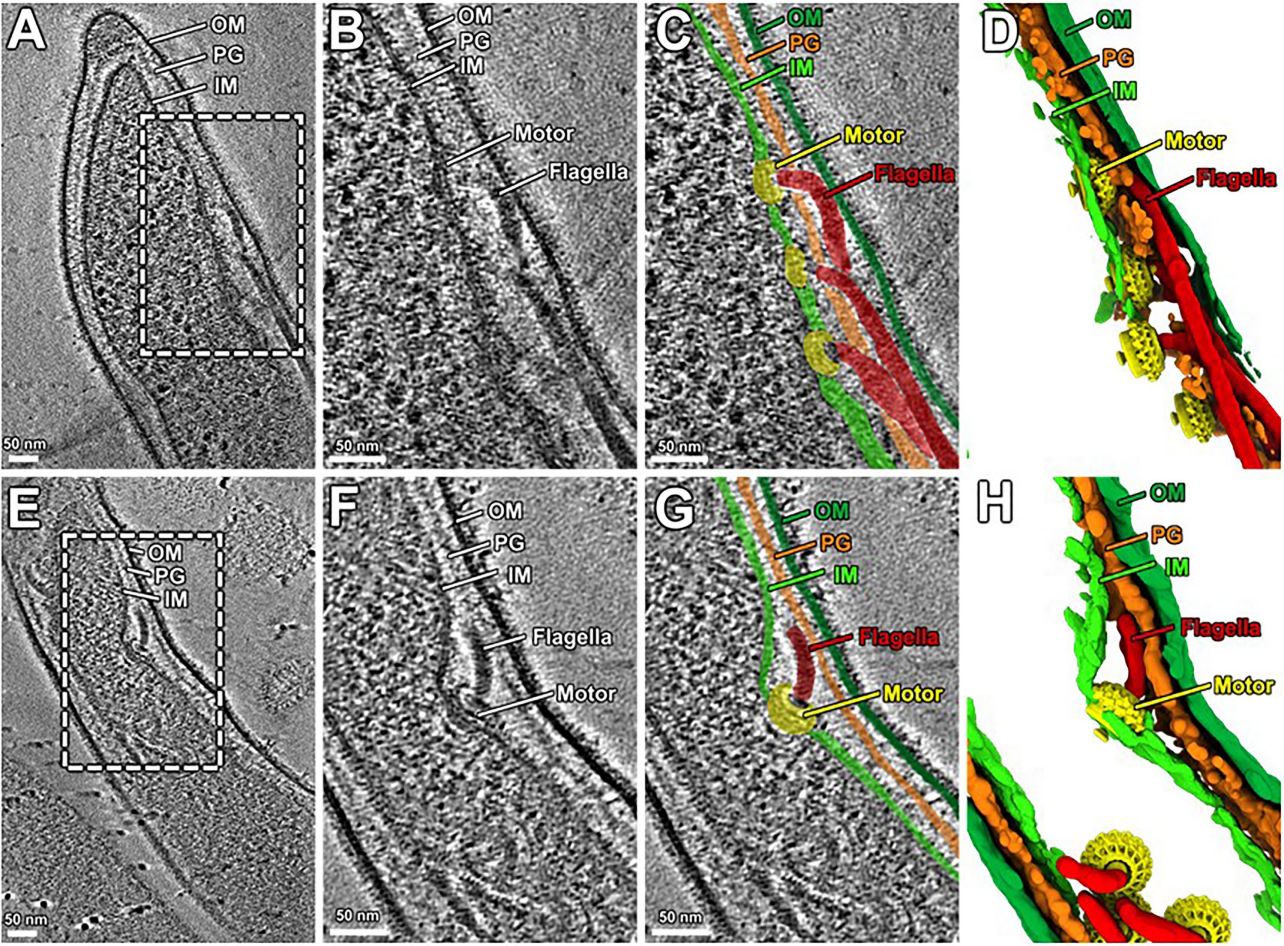
Cryo-ET analysis of *bb0259*-D606N and *bb0259*-E580Q. **(A)** A representative tomographic slice from a *bb0259*-D606N cell tip shows multiple intact flagella, including the motor, hook, and the filament. **(B)** A zoom-in view. **(C)** The motors are colored in yellow, and the hooks and filaments are in red. The inner membrane (light green), outer membrane (dark green), and PG (orange) are colored differently. **(D)** A 3D view shows the motors (yellow) and the hooks and filaments (red). **(E)** A representative tomographic slice from a *bb0259*-E580Q cell tip shows multiple flagella without filament. **(F)** A zoom-in view of one motor and its hook. **(G)** The motor is colored in yellow, and the hook is in red. **(H)** A 3D view shows the motors (yellow) and the hooks (red).

**Figure 4:**
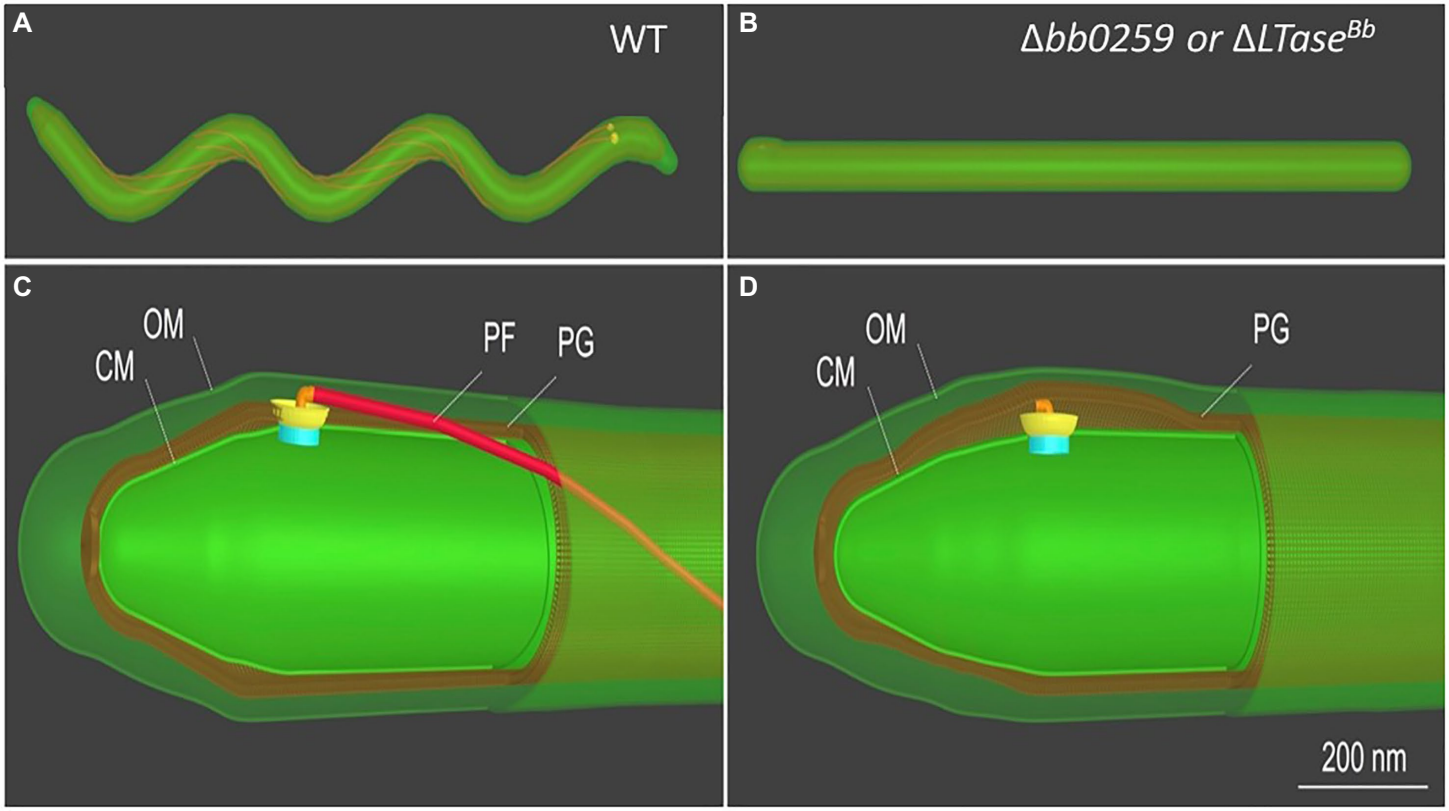
Schematic of *B. burgdorferi* WT and Δ*bb0259* mutant cells. *B. burgdorferi* cells summarizing the results from Figure 3. Δ*bb0259* is alternatively called *ΔLTase^Bb^*. **(A)** A WT *B. burgdorferi* cell has a flat-wave shape with multiple periplasmic flagella, which reside and rotate in the periplasmic space. **(B)** A *ΔLTase^Bb^* mutant cell is rod-shaped because of the lack of flagellar filaments. **(C)** A zoom-in view of the WT cell tip shows one motor and its long flagellar filament in the periplasmic space as its hook was able to penetrate the PG sacculus to complete assembly of an intact flagellum. **(D)** A zoom-in view of the *ΔLTase^Bb^* mutant cell shows a hook underneath the PG layer lacking the filament as its hook failed to penetrate the cell wall.

The morphology and motility phenotypes of the mutant cells led us to speculate that the periplasmic flagellar rod/hook/filament is not able to assemble properly due to the lack of PG-hydrolyzing activity of the Δ*bb0259* and *bb0259*-E580Q cells. Despite using the best possible assays to determine whether BB0259 is able to hydrolyze PG, as this protein possesses an SLT domain, our numerous attempts to demonstrate such activity resulted in little success. Attempts to determine the PG-hydrolyzing enzyme assays using various published assay conditions were unsuccessful even when utilizing the purified *B. burgdorferi* PG as a substrate (Jutras et al., 2016). As an alternative to enzyme assays, we used cryo-electron tomography (cryo-ET) to directly visualize the mutant’s flagella *in situ* to determine whether the rod/hook/filament assembles or whether the flagella are able to penetrate the PG-layer in the mutant cells. As shown in cryo-ET images (Supplementary Figure S4), the Δ*bb0259* mutant cells appear able to synthesize or assemble the rod, P-ring, and hook structures but lack the filament structure (Supplementary Figures S4A,B). The hook length of the Δ*bb0259* mutant cells was measured to be the same as that of wild-type cells (~51nm; *n*=20), and the hooks of the mutant cells look to be underneath the peptidoglycan layer (Figures 3E–H, 4D). However, similar cryo-ET data from the wild-type and complemented *bb0259^com^* cell tips show the intact rod, P-ring, and hook as well as filament (Supplementary Figures S4D,E,G). The averaged motor structure from the mutant cells appears to be identical to the wild-type motor structure, without any visible defect in the rod, P-ring, stator, C-ring, export apparatus, or collar structure (Supplementary Figures S4C,F). Importantly, the hooks from the *bb0259*-E580Q or Δ*bb0259* cells were found to be underneath the peptidoglycan layer (Figures 3E–H, 4D), whereas an intact rod-hook–filament structure was clearly assembled in the wild-type, *bb0259*-D606N point mutant, and complemented *bb0259^com^* cells (Figures 3A–D, 4C). The Δ*bb0259* or *bb0259*-E580Q mutant completed assembly of all the components of the flagella except the filament, and its hooks were not able to penetrate through the PG (Figures 3E–H, 4D). These results suggest that BB0259 possesses PG-lytic activity; that residue E580, but not D606, is essential for the catalytic activity of the enzyme; and that hydrolyzing the PG sacculus to allow the hook to penetrate through the cell wall is essential for completion of the assembly of an intact flagellum. We therefore renamed the BB0259 protein flagellar-specific LTase^Bb^. On the other hand, cryo-ET images of the Δ*bb0531* mutant cells show that the periplasmic flagella are completely assembled like the wild-type cells without any detectable defect in penetrating the PG layer or assembly of the flagella indicating that BB0531 is not involved in PG hydrolytic activity for flagellar penetration (Supplementary Figure S6).

### BB0259 or LTase^Bb^ Interacts With the Single-Domain Protein FlgJ

**Figure 5:**
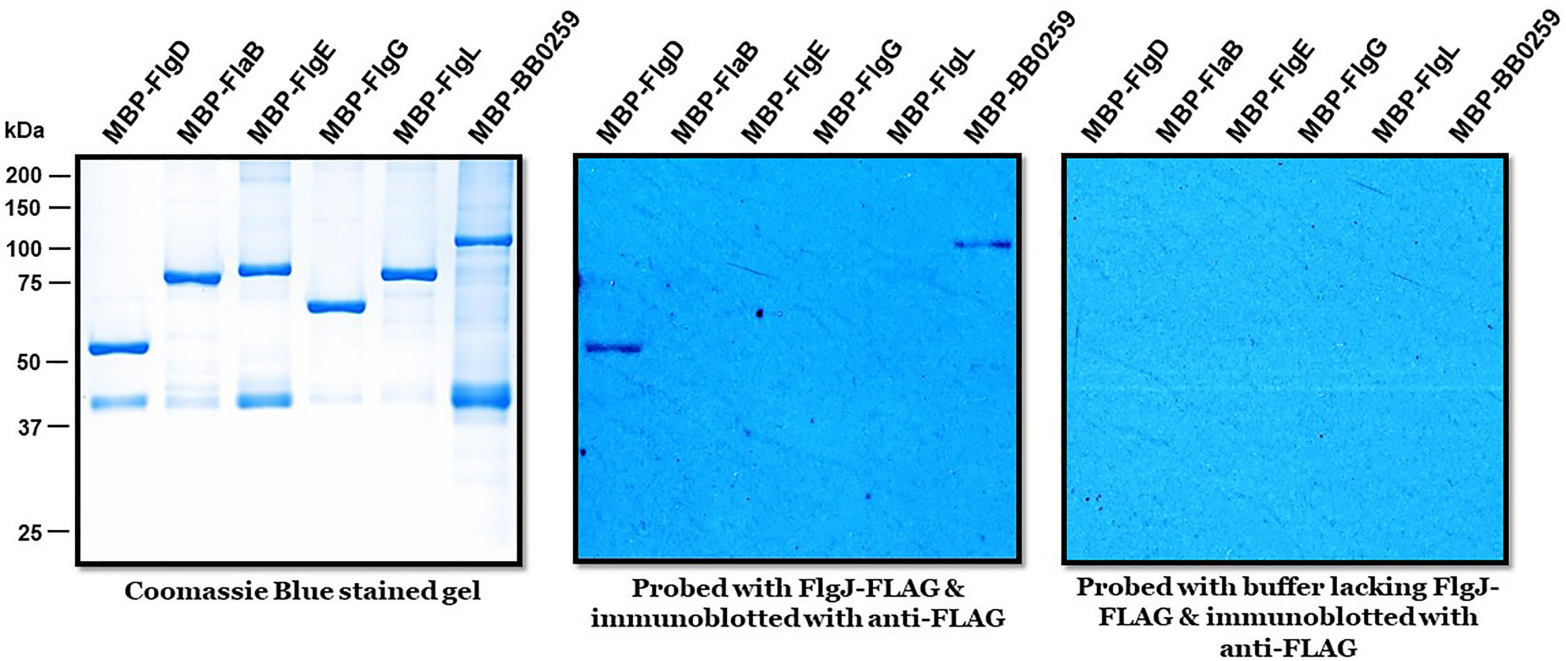
Biophysical interactions of FlgJ with BB0259/LTase^Bb^ and FlgD. Approximately 1μg of maltose binding protein (MBP)-tagged proteins shown on top of each panel were subjected to sodium dodecyl sulfate–polyacrylamide gel electrophoresis and stained with Coomassie blue (left) or transferred to a polyvinylidene difluoride membrane (middle & right panels). The membranes were incubated with or without FLAG-FlgJ and then immunoblotted with anti-FLAG monoclonal antibodies.

The requirement of PG-hydrolyzing activity for proper assembly of periplasmic flagella and consequently for motility suggests that LTase^Bb^ is essential for creating space within the peptidoglycan sacculus for insertion of cell-envelope spanning structures such as flagella. Given that the single-domain FlgJ protein lacks a PG-hydrolyzing enzyme domain, LTase^Bb^ is postulated to interact with FlgJ as the enzyme creates pores in the PG sacculus for the hook to assemble through the layer, subsequently allowing the completion of hook–filament structures for cells to be mobile. To test this proposition or to determine which proteins FlgJ interacts with, we performed far-western or affinity blotting. Recombinant FlgJ protein was used as a probe to incubate a PVDF membrane transferred with the distal rod protein FlgG, hook protein FlgE, hook cap protein FlgD, hook–filament junction protein FlgL, filament protein FlaB, and LTase^Bb^. As shown in Figure 5, FlgJ specifically interacts with LTase^Bb^ and the hook cap protein FlgD.

## Discussion

Soluble lytic transglycosylases cleave the glycosidic linkage between N-actetylmuramoyl and N-acetylglucosaminyl residues with the simultaneous production of a 1,6-anydromuramoyl product (Holtje et al., 1975; Herlihey et al., 2014). These enzymes, transglycosylases, are abundant in bacteria, found in different forms, and crucial for generating pores by hydrolyzing the PG sacculus for biosynthesis and recycling of PG, bacterial cell division, and the insertion of cell membrane-spanning structures such as flagella (Herlihey et al., 2014). *B. burgdorferi* LTase^Bb^ appears similar to the *S. enterica* and *E. coli* SLT proteins as it possesses the conserved ES-GLMQ-AYNAG motif, with E580 being the catalytic residue (Figure 1). Furthermore, the C-terminal domain with the enzymatic motif encompassing the amino acid residues 559-717 is also similar in size to the PG-hydrolytic domains of other bacteria (de la Mora et al., 2012). While a confirmatory enzymatic assay is required to state whether BB0259 in fact possesses any enzymatic activity, our numerous attempts at such an assay were unsuccessful. It is noteworthy that measuring the peptidoglycan-degrading or lytic activity of FlgJ/SLT proteins poses significant technical drawbacks. The lack of a defined substrate, the heterogeneity of both the substrate and resulting hydrolyzing products in terms of peptide chain composition and extent of PG sacculus cross-linking, and any modification to the glycan chains contribute to the complexity of analysis of the peptidoglycan. However, genetic studies concomitant with direct visualization of the flagella *in situ* by cryo-ET enabled us to propose that BB0259 possesses peptidoglycan pore-forming activities and that E580 is the catalytic residue for the peptidoglycan lytic activities, as the *bb0259*-E580Q mutant’s hooks were unable to penetrate the peptidoglycan sacculus (Figure 3).

**Figure 6:**
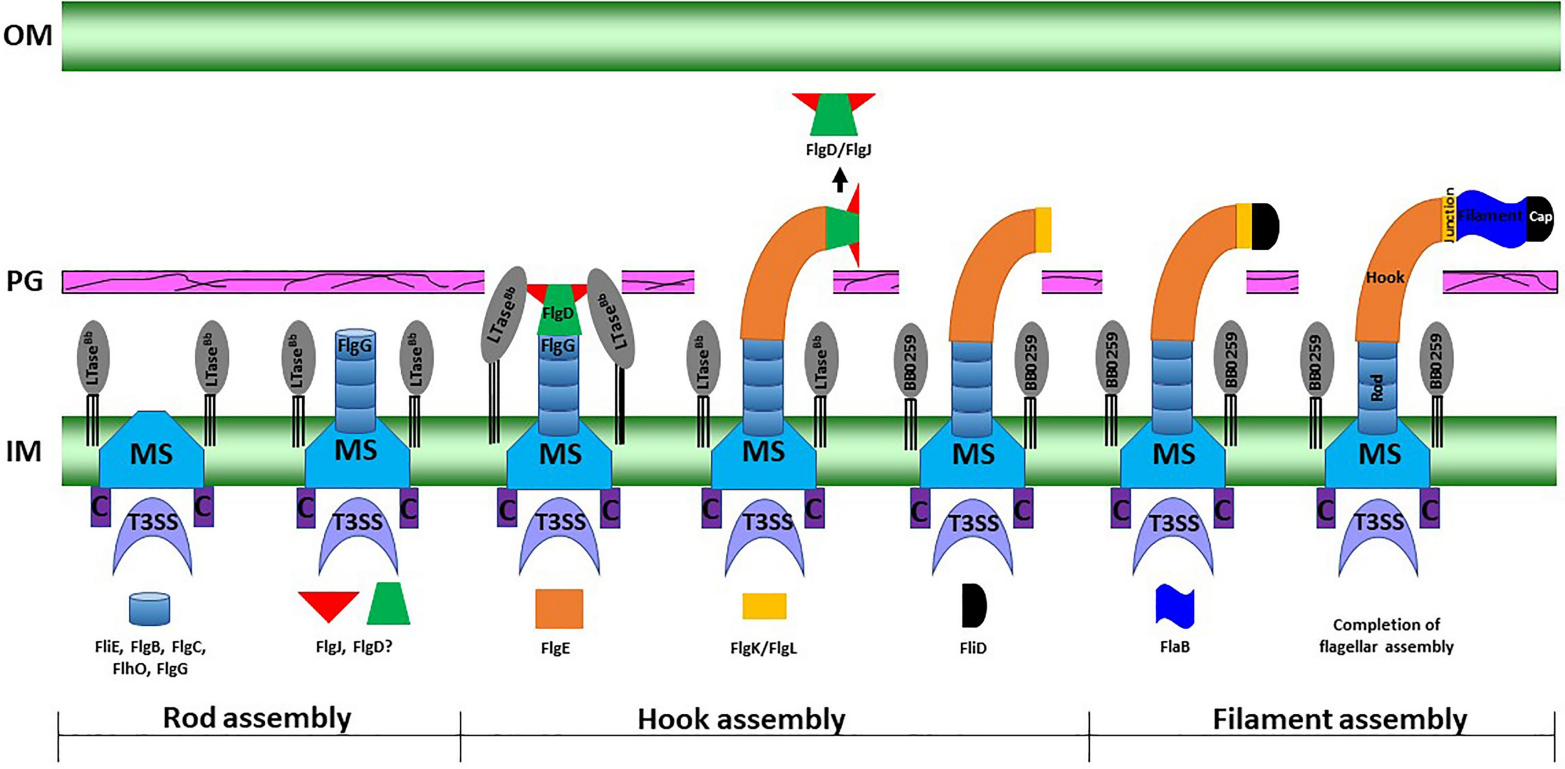
A working model of periplasmic flagellar hook–filament assembly in the spirochete *B. burgdorferi*. At the onset of rod assembly, many flagellar components assemble, such as the MS ring, C ring, export apparatus, stators, and collar. Subsequently, the rod components (FliE, FlgB, FlgC, FlhO/FlgF, and FlgG) are sequentially secreted into the periplasmic space through flagellar T3SS and assemble (Zhao et al., 2013). The hook cap protein FlgD and its chaperone or interacting protein FlgJ form at the distal end of the rod and initiate hook assembly. Binding of FlgJ with the pre-localized SLT enzyme BB0259 or LTase^Bb^ in the periplasmic leaflet of the cell wall promotes hydrolysis of the sacculus as the hook completes assembly through the cell wall. Assembly of the filament (FlaA and FlaB) is promoted by the filament cap.

LTase^Bb^ possesses an N-terminal peptide with unknown function (25-558 aa). As the C-terminal domain possesses the enzymatic motif, we speculate that the N-terminal domain is involved in binding to FlgJ, as LTase^Bb^ secretes into the periplasm using its Sec-signaling sequence. Since LTase^Bb^ appears to possess peptidoglycan lytic activities and FlgG and FlgJ are required for proper rod/hook/filament assembly (Zhang et al., 2012; Zhao et al., 2013), we propose that these proteins physically interact to facilitate the insertion of the flagella through the PG layer as the enzyme creates pores in the sacculus (Figure 6). Our direct visualization of the mutant’s flagella *in situ* by cryo-ET shows that the hooks are unable to pass through the PG sacculus, and therefore, these structures were detected just underneath the PG layer (Figures 3E–H, 4D). Consequently, the flagella did not assemble properly, leading to the non-motile and rod-shaped morphology (Figures 2A,B).

*Borrelia burgdorferi* FlgJ and the SLT domain-containing LTase^Bb^ are distinct from their counterparts in *S. enterica*. For example, FlgJ from *S. enterica* is involved in flagellar rod formation or required for the transition from rod to hook formation (Cohen and Hughes, 2014). However, cryo-ET data from the *B. burgdorferi* Δ*flgJ* mutant show that the mutant completed assembly of the rod and P-ring but assembled a partial hook and filament structures (Zhang et al., 2012). It is noteworthy that, unlike the model organisms, the rod and P-ring in this spirochete are not able to contact the PG layer, as the rod is significantly smaller than in the model organisms (17nm vs. 30nm in *E. coli*; Zhao et al., 2013; Saijo-Hamano et al., 2019). It is the spirochete-specific collar that contacts the PG sacculus, while the hook penetrates the peptidoglycan layer to complete assembly and formation of the filament in *B. burgdorferi* (Zhao et al., 2013). FlgJ in the spirochete is therefore likely important for assembly of the hook/filament during the hook-to-filament transition (Zhang et al., 2012). The mutants of *bb0259* (or *LTase^Bb^*) also assemble the rod, P-ring, and hook but are unable to synthesize/assemble the filament (Figures 3, 4, Supplementary Figure S3). The hooks are not able to penetrate the PG layer due to the lack of PG lytic activity in Δ*bb0259* or *bb0259*-E580Q mutants (Δ*LTase^Bb^* or *LTase^Bb^*-E580Q, respectively). This result also enforces the notion that FlgJ and LTase^Bb^ are crucial for hook/ filament assembly and/or for hook-to-filament transition (Figure 6).

Δ*bb0259/*Δ*LTase^Bb^* or *bb0259*-E580Q/*LTase^Bb^*-E580Q mutant phenotypes appear to be very similar to that of the Δ*flaB* mutant (Zhao et al., 2013). In both cases, the mutants complete assembly and formation of the motor, rod, P-ring, and hook but lack the filament (compare Figures 3F,G with Supplementary Figure S4H or Figure 5A from ref. Zhao et al., 2013). However, in the *flaB* mutant, the hook is able to penetrate the PG layer (Supplementary Figure S4H) as the PG enzyme activity of BB0259 is believed to be retained in Δ*flaB* cells, again reinforcing that BB0259 or LTase^Bb^ is a PG hydrolyzing enzyme. Moreover, the hook length of the Δ*LTase^Bb^*, Δ*flaB*, and wild-type cells is the same (~51nm; *n*=20) and consistent with the previously reported hook length (Zhao et al., 2013), suggesting that LTase^Bb^ is not involved in hook assembly. All these data suggest that FlgJ and LTase^Bb^ are distinct from their counterparts in other flagellated bacteria reported to date. Importantly, in *S. enterica*, FlgJ interacts with the rod proteins including the distal rod FlgG as FlgJ is considered a rod-capping or scaffolding protein (Hirano et al., 2001; Cohen and Hughes, 2014). However, that is not the case in *B. burgdorferi*. FlgJ interaction with FlgG is undetectable (Figure 5), and the Δ*flgJ* mutant’s rod structures are intact in *B. burgdorferi* (Zhang et al., 2012).

Based on reports of FlgJ and this communication, we propose a model for how LTase^Bb^ and FlgJ work synergistically with the hook cap protein FlgD to complete assembly of the hook–filament in *B. burgdorferi* (Figure 6). In this model, LTase^Bb^ uses Sec-dependent pathway to be secreted into the periplasm. Using the flagellar type III secretion system, FlgJ and hook-capping protein FlgD compile onto the rod as the rod completes assembly onto the MS-ring. Interactions of FlgJ with BB0259 or LTase^Bb^ and FlgD (Figure 5) enable the hook to initiate and complete its assembly through the PG holes created by LTase^Bb^ wherein FlgJ chaperones or interacts with FlgD for the hook to complete its assembly in the periplasm (Figure 6). Without the FlgJ, the hook FlgE synthesis or assembly is therefore incomplete (Zhang et al., 2012) due to a lack of interaction between FlgD and FlgJ or lack of chaperone functions of FlgJ for FlgD. We speculate that the hook-to-filament assembly occurs as in other bacteria, as we have reported (Zhao et al., 2013). Additional in-depth analysis is warranted to better understand the spatiotemporal synthesis of the FlgJ, FlgD, and LTase^Bb^ proteins and hook–filament assemblies in *B. burgdorferi*.

## Data Availability Statement

The raw data supporting the conclusions of this article will be made available by the authors, without undue reservation.

## Author Contributions

HX, BH, and DAF conducted the experiments. JL and MM designed the experiments. All authors contributed to the article and approved the submitted version.

## Funding

JL was supported by grants GM107629 and R01AI087946. MM was supported by R01AI132818 from National Institutes of Health (NIH). Part of cryo-ET data were collected at Yale CryoEM resource that is funded in part by the NIH grant 1S10OD023603-01A1.

## Conflict of Interest

The authors declare that the research was conducted in the absence of any commercial or financial relationships that could be construed as a potential conflict of interest.

## Publisher’s Note

All claims expressed in this article are solely those of the authors and do not necessarily represent those of their affiliated organizations, or those of the publisher, the editors and the reviewers. Any product that may be evaluated in this article, or claim that may be made by its manufacturer, is not guaranteed or endorsed by the publisher.

## Footnotes

^1^ https://ffas.godziklab.org/ffas-cgi/cgi/ffas.pl

## References

[ref1] Almagro Armenteros J. J. Tsirigos K. D. Sonderby C. K. Petersen T. N. Winther O. Brunak S.. (2019). SignalP 5.0 improves signal peptide predictions using deep neural networks. Nat. Biotechnol. 37, 420–423. doi: 10.1038/s41587-019-0036-z, PMID: 30778233

[ref2] Altschul S. F. Gish W. Miller W. Myers E. W. Lipman D. J. (1990). Basic local alignment search tool. J. Mol. Biol. 215, 403–410. doi: 10.1016/S0022-2836(05)80360-2, PMID: 2231712

[ref3] Altschul S. F. Madden T. L. Schaffer A. A. Zhang J. Zhang Z. Miller W.. (1997). Gapped BLAST and PSI-BLAST: a new generation of protein database search programs. Nucleic Acids Res. 25, 3389–3402. doi: 10.1093/nar/25.17.3389, PMID: 9254694 PMC146917

[ref4] Babu M. M. Priya M. L. Selvan A. T. Madera M. Gough J. Aravind L.. (2006). A database of bacterial lipoproteins (DOLOP) with functional assignments to predicted lipoproteins. J. Bacteriol. 188, 2761–2773. doi: 10.1128/JB.188.8.2761-2773.2006, PMID: 16585737 PMC1446993

[ref5] Bertani G. (1951). Studies on lysogenesis. I. The mode of phage liberation by lysogenic *Escherichia coli*. J. Bacteriol. 62, 293–300. doi: 10.1128/jb.62.3.293-300.1951, PMID: 14888646 PMC386127

[ref6] Bertani G. (2004). Lysogeny at mid-twentieth century: P1, P2, and other experimental systems. J. Bacteriol. 186, 595–600. doi: 10.1128/JB.186.3.595-600.2004, PMID: 14729683 PMC321500

[ref7] Blackburn N. T. Clarke A. J. (2001). Identification of four families of peptidoglycan lytic transglycosylases. J. Mol. Evol. 52, 78–84. doi: 10.1007/s002390010136, PMID: 11139297

[ref8] Bono J. L. Elias A. F. Kupko J. J. 3rd Stevenson B. Tilly K. Rosa P. (2000). Efficient targeted mutagenesis in *Borrelia burgdorferi*. J. Bacteriol. 182, 2445–2452. doi: 10.1128/JB.182.9.2445-2452.2000, PMID: 10762244 PMC111306

[ref9] Carroll B. L. Liu J. (2020). Structural conservation and adaptation of the bacterial flagella motor. Biomol. Ther. 10:1492. doi: 10.3390/biom10111492, PMID: 33138111 PMC7693769

[ref10] Charon N. W. Cockburn A. Li C. Liu J. Miller K. A. Miller M. R.. (2012). The unique paradigm of spirochete motility and chemotaxis. Annu. Rev. Microbiol. 66, 349–370. doi: 10.1146/annurev-micro-092611-150145, PMID: 22994496 PMC3771095

[ref11] Charon N. W. Goldstein S. F. Marko M. Hsieh C. Gebhardt L. L. Motaleb M. A.. (2009). The flat ribbon configuration of the periplasmic flagella of *Borrelia burgdorferi* and its relationship to motility and morphology. J. Bacteriol. 191, 600–607. doi: 10.1128/JB.01288-08, PMID: 19011030 PMC2620816

[ref12] Chen S. Beeby M. Murphy G. E. Leadbetter J. R. Hendrixson D. R. Briegel A.. (2011). Structural diversity of bacterial flagellar motors. EMBO J. 30, 2972–2981. doi: 10.1038/emboj.2011.186, PMID: 21673657 PMC3160247

[ref13] Chen M. Dai W. Sun S. Y. Jonasch D. He C. Y. Schmid M. F.. (2017). Convolutional neural networks for automated annotation of cellular cryo-electron tomograms. Nat. Methods 14, 983–985. doi: 10.1038/nmeth.4405, PMID: 28846087 PMC5623144

[ref14] Cohen E. J. Hughes K. T. (2014). Rod-to-hook transition for extracellular flagellum assembly is catalyzed by the L-ring-dependent rod scaffold removal. J. Bacteriol. 196, 2387–2395. doi: 10.1128/JB.01580-14, PMID: 24748615 PMC4054162

[ref15] de la Mora J. Osorio-Valeriano M. Gonzalez-Pedrajo B. Ballado T. Camarena L. Dreyfus G. (2012). The C terminus of the flagellar muramidase SltF modulates the interaction with FlgJ in *Rhodobacter sphaeroides*. J. Bacteriol. 194, 4513–4520. doi: 10.1128/JB.00460-12, PMID: 22707709 PMC3415505

[ref16] Demchick P. Koch A. L. (1996). The permeability of the wall fabric of *Escherichia coli* and *Bacillus subtilis*. J. Bacteriol. 178, 768–773. doi: 10.1128/jb.178.3.768-773.1996, PMID: 8550511 PMC177723

[ref17] Elias A. F. Bono J. L. Kupko J. J. 3rd Stewart P. E. Krum J. G. Rosa P. A. (2003). New antibiotic resistance cassettes suitable for genetic studies in *Borrelia burgdorferi*. J. Mol. Microbiol. Biotechnol. 6, 29–40. doi: 10.1159/000073406, PMID: 14593251

[ref18] Garcia-Ramos M. de la Mora J. Ballado T. Camarena L. Dreyfus G. (2018). Biochemical and phylogenetic study of SltF, a flagellar lytic transglycosylase from *Rhodobacter sphaeroides*. J. Bacteriol. 200:e00397-18. doi: 10.1128/JB.00397-18, PMID: 30061356 PMC6153662

[ref19] Ge Y. Old I. G. Saint Girons I. Charon N. W. (1997). Molecular characterization of a large *Borrelia burgdorferi* motility operon which is initiated by a consensus sigma70 promoter. J. Bacteriol. 179, 2289–2299. doi: 10.1128/jb.179.7.2289-2299.1997, PMID: 9079915 PMC178966

[ref20] Goddard T. D. Huang C. C. Meng E. C. Pettersen E. F. Couch G. S. Morris J. H.. (2018). UCSF ChimeraX: Meeting modern challenges in visualization and analysis. Protein Sci. 27, 14–25. doi: 10.1002/pro.3235, PMID: 28710774 PMC5734306

[ref21] Gonzalez-Pedrajo B. de la Mora J. Ballado T. Camarena L. Dreyfus G. (2002). Characterization of the flgG operon of *Rhodobacter sphaeroides* WS8 and its role in flagellum biosynthesis. Biochim. Biophys. Acta 1579, 55–63. doi: 10.1016/s0167-4781(02)00504-3, PMID: 12401220

[ref22] Herlihey F. A. Moynihan P. J. Clarke A. J. (2014). The essential protein for bacterial flagella formation FlgJ functions as a beta-N-acetylglucosaminidase. J. Biol. Chem. 289, 31029–31042. doi: 10.1074/jbc.M114.603944, PMID: 25248745 PMC4223308

[ref23] Hirano T. Minamino T. Macnab R. M. (2001). The role in flagellar rod assembly of the N-terminal domain of Salmonella FlgJ, a flagellum-specific muramidase. J. Mol. Biol. 312, 359–369. doi: 10.1006/jmbi.2001.4963, PMID: 11554792

[ref24] Holtje J. V. Mirelman D. Sharon N. Schwarz U. (1975). Novel type of murein transglycosylase in *Escherichia coli*. J. Bacteriol. 124, 1067–1076. doi: 10.1128/jb.124.3.1067-1076.1975, PMID: 357 PMC236007

[ref25] Homma M. Iino T. (1985). Locations of hook-associated proteins in flagellar structures of *Salmonella typhimurium*. J. Bacteriol. 162, 183–189. doi: 10.1128/jb.162.1.183-189.1985, PMID: 3884587 PMC218972

[ref26] Jaroszewski L. Li Z. Cai X. H. Weber C. Godzik A. (2011). FFAS server: novel features and applications. Nucleic Acids Res. 39, W38–W44. doi: 10.1093/nar/gkr441, PMID: 21715387 PMC3125803

[ref27] Jaroszewski L. Rychlewski L. Li Z. Li W. Godzik A. (2005). FFAS03: a server for profile–profile sequence alignments. Nucleic Acids Res. 33, W284–W288. doi: 10.1093/nar/gki418, PMID: 15980471 PMC1160179

[ref28] Jones C. J. Macnab R. M. (1990). Flagellar assembly in *Salmonella typhimurium*: analysis with temperature-sensitive mutants. J. Bacteriol. 172, 1327–1339. doi: 10.1128/jb.172.3.1327-1339.1990, PMID: 2407720 PMC208602

[ref29] Jutras B. L. Scott M. Parry B. Biboy J. Gray J. Vollmer W.. (2016). Lyme disease and relapsing fever Borrelia elongate through zones of peptidoglycan synthesis that mark division sites of daughter cells. Proc. Natl. Acad. Sci. U. S. A. 113, 9162–9170. doi: 10.1073/pnas.1610805113, PMID: 27506799 PMC4995948

[ref30] Kall L. Krogh A. Sonnhammer E. L. (2004). A combined transmembrane topology and signal peptide prediction method. J. Mol. Biol. 338, 1027–1036. doi: 10.1016/j.jmb.2004.03.016, PMID: 15111065

[ref31] Kall L. Krogh A. Sonnhammer E. L. (2005). An HMM posterior decoder for sequence feature prediction that includes homology information. Bioinformatics 21(Suppl. 1), i251–i257. doi: 10.1093/bioinformatics/bti1014, PMID: 15961464

[ref32] Kall L. Krogh A. Sonnhammer E. L. (2007). Advantages of combined transmembrane topology and signal peptide prediction–the Phobius web server. Nucleic Acids Res. 35, W429–W432. doi: 10.1093/nar/gkm256, PMID: 17483518 PMC1933244

[ref33] Kariu T. Sharma K. Singh P. Smith A. A. Backstedt B. Buyuktanir O.. (2015). BB0323 and novel virulence determinant BB0238: *Borrelia burgdorferi* proteins that interact with and stabilize each other and are critical for infectivity. J. Infect. Dis. 211, 462–471. doi: 10.1093/infdis/jiu460, PMID: 25139020 PMC4351374

[ref34] Koraimann G. (2003). Lytic transglycosylases in macromolecular transport systems of Gram-negative bacteria. Cell. Mol. Life Sci. 60, 2371–2388. doi: 10.1007/s00018-003-3056-1, PMID: 14625683 PMC11138577

[ref35] Kremer J. R. Mastronarde D. N. McIntosh J. R. (1996). Computer visualization of three-dimensional image data using IMOD. J. Struct. Biol. 116, 71–76. doi: 10.1006/jsbi.1996.0013, PMID: 8742726

[ref36] Li C. Xu H. Zhang K. Liang F. T. (2010). Inactivation of a putative flagellar motor switch protein FliG1 prevents *Borrelia burgdorferi* from swimming in highly viscous media and blocks its infectivity. Mol. Microbiol. 75, 1563–1576. doi: 10.1111/j.1365-2958.2010.07078.x, PMID: 20180908 PMC4394363

[ref37] Liu J. Lin T. Botkin D. J. McCrum E. Winkler H. Norris S. J. (2009). Intact flagellar motor of *Borrelia burgdorferi* revealed by cryo-electron tomography: evidence for stator ring curvature and rotor/C-ring assembly flexion. J. Bacteriol. 191, 5026–5036. doi: 10.1128/JB.00340-09, PMID: 19429612 PMC2725586

[ref38] Madan Babu M. Sankaran K. (2002). DOLOP–database of bacterial lipoproteins. Bioinformatics 18, 641–643. doi: 10.1093/bioinformatics/18.4.641, PMID: 12016064

[ref39] Moon K. H. Hobbs G. Motaleb M. A. (2016). *Borrelia burgdorferi* CheD promotes various functions in chemotaxis and the pathogenic life cycle of the spirochete. Infect. Immun. 84, 1743–1752. doi: 10.1128/IAI.01347-15, PMID: 27021244 PMC4907153

[ref40] Moon K. H. Zhao X. Xu H. Liu J. Motaleb M. A. (2018). A tetratricopeptide repeat domain protein has profound effects on assembly of periplasmic flagella, morphology and motility of the Lyme disease spirochete *Borrelia burgdorferi*. Mol. Microbiol. 110, 634–647. doi: 10.1111/mmi.14121, PMID: 30303576 PMC6218285

[ref41] Motaleb M. A. Corum L. Bono J. L. Elias A. F. Rosa P. Samuels D. S.. (2000). *Borrelia burgdorferi* periplasmic flagella have both skeletal and motility functions. Proc. Natl. Acad. Sci. U. S. A. 97, 10899–10904. doi: 10.1073/pnas.200221797, PMID: 10995478 PMC27121

[ref42] Motaleb M. A. Liu J. Wooten R. M. (2015). Spirochetal motility and chemotaxis in the natural enzootic cycle and development of Lyme disease. Curr. Opin. Microbiol. 28, 106–113. doi: 10.1016/j.mib.2015.09.006, PMID: 26519910 PMC4688064

[ref43] Motaleb M. A. Miller M. R. Bakker R. G. Li C. Charon N. W. (2007). Isolation and characterization of chemotaxis mutants of the Lyme disease Spirochete *Borrelia burgdorferi* using allelic exchange mutagenesis, flow cytometry, and cell tracking. Methods Enzymol. 422, 421–437. doi: 10.1016/S0076-6879(06)22021-4, PMID: 17628152

[ref44] Motaleb M. A. Pitzer J. E. Sultan S. Z. Liu J. (2011). A novel gene inactivation system reveals altered periplasmic flagellar orientation in a *Borrelia burgdorferi* fliL mutant. J. Bacteriol. 193, 3324–3331. doi: 10.1128/JB.00202-11, PMID: 21441522 PMC3133274

[ref45] Nakamura S. Minamino T. (2019). Flagella-driven motility of bacteria. Biomol. Ther. 9:279. doi: 10.3390/biom9070279, PMID: 31337100 PMC6680979

[ref46] Nambu T. Inagaki Y. Kutsukake K. (2006). Plasticity of the domain structure in FlgJ, a bacterial protein involved in flagellar rod formation. Genes Genet. Syst. 81, 381–389. doi: 10.1266/ggs.81.381, PMID: 17283383

[ref47] Nambu T. Minamino T. Macnab R. M. Kutsukake K. (1999). Peptidoglycan-hydrolyzing activity of the FlgJ protein, essential for flagellar rod formation in *Salmonella typhimurium*. J. Bacteriol. 181, 1555–1561. doi: 10.1128/JB.181.5.1555-1561.1999, PMID: 10049388 PMC93546

[ref48] Ohnishi K. Ohto Y. Aizawa S. I. Macnab R. M. Iino T. (1994). FlgD is a scaffolding protein needed for flagellar hook assembly in *Salmonella typhimurium*. J. Bacteriol. 176, 2272–2281. doi: 10.1128/jb.176.8.2272-2281.1994, PMID: 8157595 PMC205349

[ref49] Rychlewski L. Jaroszewski L. Li W. Godzik A. (2000). Comparison of sequence profiles. Strategies for structural predictions using sequence information. Protein Sci. 9, 232–241. doi: 10.1110/ps.9.2.232, PMID: 10716175 PMC2144550

[ref50] Saijo-Hamano Y. Matsunami H. Namba K. Imada K. (2019). Architecture of the bacterial flagellar distal rod and hook of *Salmonella*. Biomol. Ther. 9:260. doi: 10.3390/biom9070260, PMID: 31284631 PMC6681337

[ref51] Sal M. S. Li C. Motalab M. A. Shibata S. Aizawa S. Charon N. W. (2008). *Borrelia burgdorferi* uniquely regulates its motility genes and has an intricate flagellar hook-basal body structure. J. Bacteriol. 190, 1912–1921. doi: 10.1128/JB.01421-07, PMID: 18192386 PMC2258876

[ref52] Sievers F. Wilm A. Dineen D. Gibson T. J. Karplus K. Li W.. (2011). Fast, scalable generation of high-quality protein multiple sequence alignments using Clustal Omega. Mol. Syst. Biol. 7:539. doi: 10.1038/msb.2011.75, PMID: 21988835 PMC3261699

[ref53] Sultan S. Z. Manne A. Stewart P. E. Bestor A. Rosa P. A. Charon N. W.. (2013). Motility is crucial for the infectious life cycle of *Borrelia burgdorferi*. Infect. Immun. 81, 2012–2021. doi: 10.1128/IAI.01228-12, PMID: 23529620 PMC3676011

[ref54] Sultan S. Z. Pitzer J. E. Miller M. R. Motaleb M. A. (2010). Analysis of a *Borrelia burgdorferi* phosphodiesterase demonstrates a role for cyclic-di-guanosine monophosphate in motility and virulence. Mol. Microbiol. 77, 128–142. doi: 10.1111/j.1365-2958.2010.07191.x, PMID: 20444101 PMC2907449

[ref55] Sultan S. Z. Sekar P. Zhao X. Manne A. Liu J. Wooten R. M.. (2015). Motor rotation is essential for the formation of the periplasmic flagellar ribbon, cellular morphology, and *Borrelia burgdorferi* persistence within *Ixodes scapularis* tick and murine hosts. Infect. Immun. 83, 1765–1777. doi: 10.1128/IAI.03097-14, PMID: 25690096 PMC4399055

[ref56] Terashima H. Kawamoto A. Morimoto Y. V. Imada K. Minamino T. (2017). Structural differences in the bacterial flagellar motor among bacterial species. Biophys. Physicobiol. 14, 191–198. doi: 10.2142/biophysico.14.0_191, PMID: 29362704 PMC5774414

[ref57] Thunnissen A. M. Dijkstra A. J. Kalk K. H. Rozeboom H. J. Engel H. Keck W.. (1994). Doughnut-shaped structure of a bacterial muramidase revealed by X-ray crystallography. Nature 367, 750–753. doi: 10.1038/367750a0, PMID: 8107871

[ref58] Tilly K. Elias A. F. Errett J. Fischer E. Iyer R. Schwartz I.. (2001). Genetics and regulation of chitobiose utilization in *Borrelia burgdorferi*. J. Bacteriol. 183, 5544–5553. doi: 10.1128/JB.183.19.5544-5553.2001, PMID: 11544216 PMC95445

[ref59] Toker A. S. Macnab R. M. (1997). Distinct regions of bacterial flagellar switch protein FliM interact with FliG, FliN and CheY. J. Mol. Biol. 273, 623–634. doi: 10.1006/jmbi.1997.1335, PMID: 9356251

[ref60] Toledo A. Huang Z. Coleman J. L. London E. Benach J. L. (2018). Lipid rafts can form in the inner and outer membranes of *Borrelia burgdorferi* and have different properties and associated proteins. Mol. Microbiol. 108, 63–76. doi: 10.1111/mmi.13914, PMID: 29377398 PMC5867248

[ref61] van Asselt E. J. Thunnissen A. M. Dijkstra B. W. (1999). High resolution crystal structures of the *Escherichia coli* lytic transglycosylase Slt70 and its complex with a peptidoglycan fragment. J. Mol. Biol. 291, 877–898. doi: 10.1006/jmbi.1999.3013, PMID: 10452894

[ref62] Wolgemuth C. W. Charon N. W. Goldstein S. F. Goldstein R. E. (2006). The flagellar cytoskeleton of the spirochetes. J. Mol. Microbiol. Biotechnol. 11, 221–227. doi: 10.1159/000094056, PMID: 16983197

[ref63] Xu H. He J. Liu J. Motaleb M. A. (2019). BB0326 is responsible for the formation of periplasmic flagellar collar and assembly of the stator complex in *Borrelia burgdorferi*. Mol. Microbiol. 113, 418–429. doi: 10.1111/mmi.14428, PMID: 31743518 PMC7178549

[ref64] Xu H. Sultan S. Yerke A. Moon K. H. Wooten R. M. Motaleb M. A. (2017). *Borrelia burgdorferi* CheY2 is dispensable for chemotaxis or motility but crucial for the infectious life cycle of the spirochete. Infect. Immun. 85:e00264-16. doi: 10.1128/IAI.00264-16, PMID: 27799336 PMC5203640

[ref65] Yang X. F. Alani S. M. Norgard M. V. (2003). The response regulator Rrp2 is essential for the expression of major membrane lipoproteins in *Borrelia burgdorferi*. Proc. Natl. Acad. Sci. U. S. A. 100, 11001–11006. doi: 10.1073/pnas.1834315100, PMID: 12949258 PMC196916

[ref66] Yonekura K. Maki S. Morgan D. G. DeRosier D. J. Vonderviszt F. Imada K.. (2000). The bacterial flagellar cap as the rotary promoter of flagellin self-assembly. Science 290, 2148–2152. doi: 10.1126/science.290.5499.2148, PMID: 11118149

[ref67] Zaloba P. Bailey-Elkin B. A. Derksen M. Mark B. L. (2016). Structural and biochemical insights into the peptidoglycan hydrolase domain of FlgJ from *Salmonella typhimurium*. PLoS One 11:e0149204. doi: 10.1371/journal.pone.0149204, PMID: 26871950 PMC4752226

[ref68] Zhang K. Tong B. A. Liu J. Li C. (2012). A single-domain FlgJ contributes to flagellar hook and filament formation in the Lyme disease spirochete *Borrelia burgdorferi*. J. Bacteriol. 194, 866–874. doi: 10.1128/JB.06341-11, PMID: 22155773 PMC3272955

[ref69] Zhao X. Zhang K. Boquoi T. Hu B. Motaleb M. A. Miller K. A.. (2013). Cryoelectron tomography reveals the sequential assembly of bacterial flagella in *Borrelia burgdorferi*. Proc. Natl. Acad. Sci. U. S. A. 110, 14390–14395. doi: 10.1073/pnas.1308306110, PMID: 23940315 PMC3761569

[ref70] Zheng S. Q. Palovcak E. Armache J. P. Verba K. A. Cheng Y. Agard D. A. (2017). MotionCor2: anisotropic correction of beam-induced motion for improved cryo-electron microscopy. Nat. Methods 14, 331–332. doi: 10.1038/nmeth.4193, PMID: 28250466 PMC5494038

