# Supplementary material for: BB0259 Encompasses a Peptidoglycan Lytic Enzyme Function for Proper Assembly of Periplasmic Flagella in Borrelia burgdorferi
Source: Front Microbiol. 2021 Oct 1;12:692707. doi: 10.3389/fmicb.2021.692707 (PMC8517470; doi:10.3389/fmicb.2021.692707)
Supplement: Supplementary file 1 [file Data_Sheet_1.PDF]

Supplemental Material

BB0259 encompasses a peptidoglycan lytic enzyme function for proper assembly of periplasmic flagella in *Borrelia burgdorferi*

Hui Xu[1], Bo Hu[2], David Flesher[3], Jun Liu[3,4,*], and Md A. Motaleb[1,*]

[1] Department of Microbiology and Immunology, Brody School of Medicine, East Carolina University, Greenville, North Carolina, United States

2 Department of Microbiology and Molecular Genetics, McGovern Medical School, The University of Texas Health Science Center at Houston, Houston, Texas, United States

[3] Department of Microbial Pathogenesis, Yale University School of Medicine, New Haven, Connecticut, United States

[4] Microbial Sciences Institute, Yale University, West Haven, Connecticut, United States.

*Correspondence: motalebm@ecu.edu; jliu@yale.edu

Figure S1

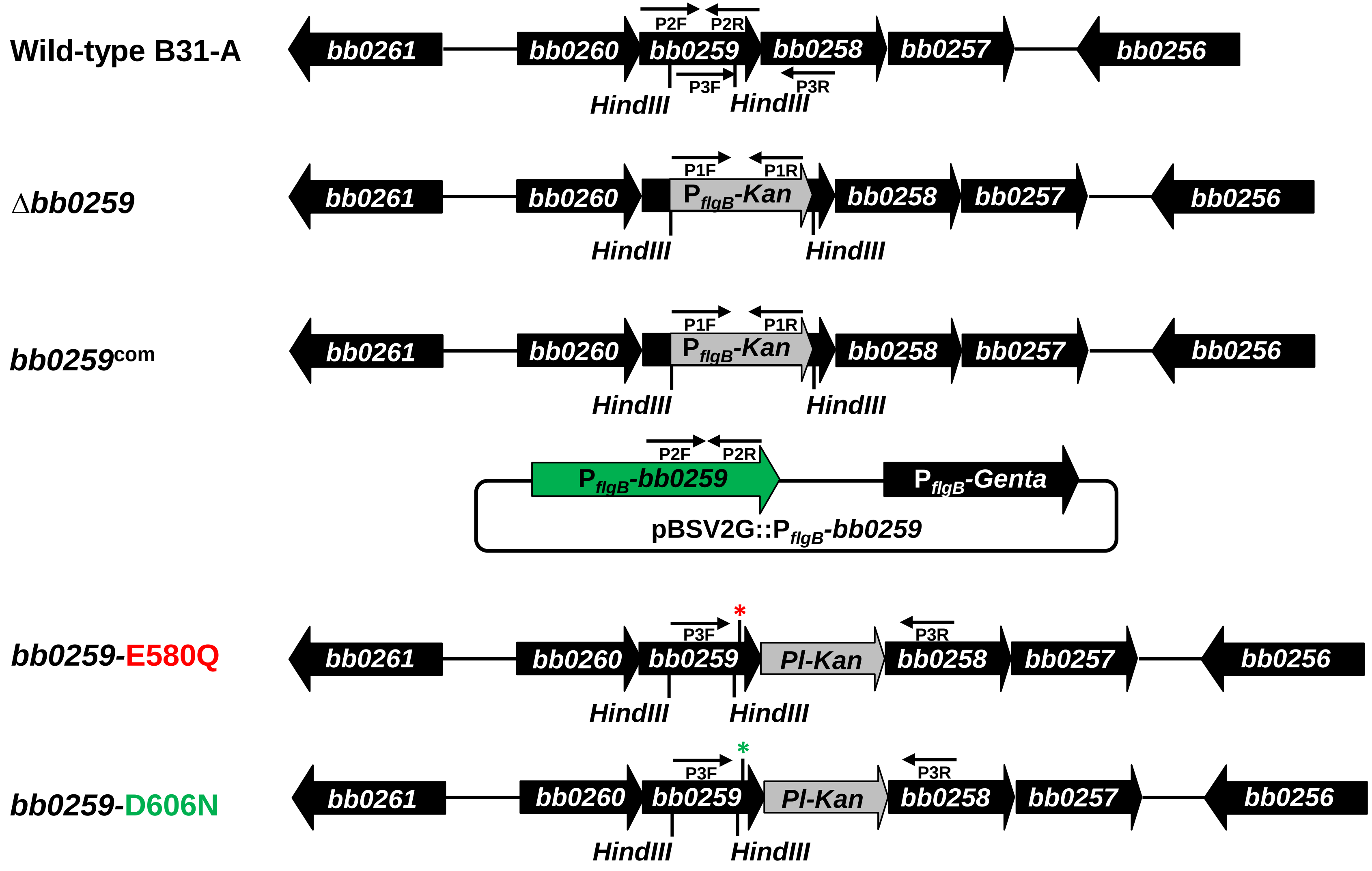

Figure S1. Inactivation, complementation and point mutations of *bb0259*. The *bb0259* gene is located within a putative operon consisting of four genes (diagram is not to scale). The diagrams illustrate the construction of each mutant strain. Details are provided in the Materials & Methods.

Figure S2

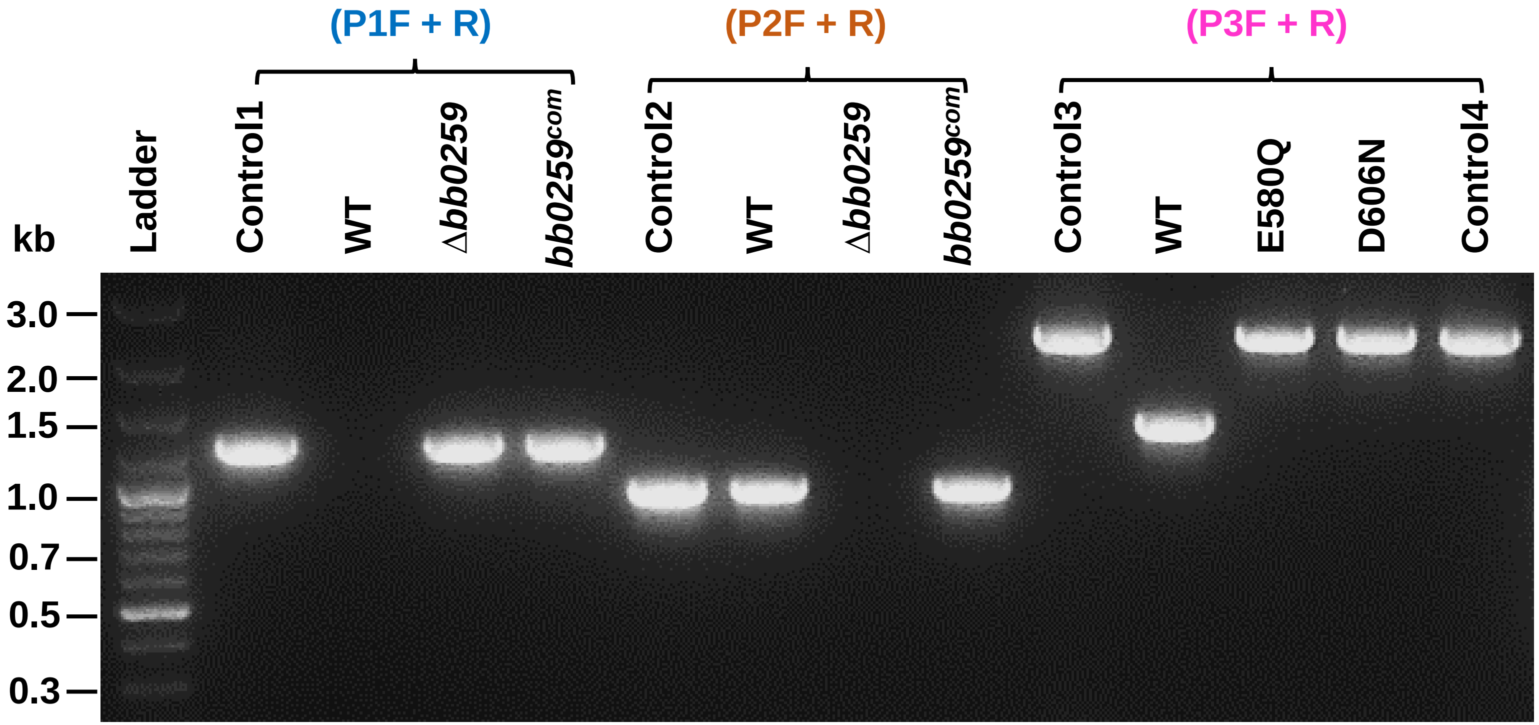

Primer name	Primer sequence	Expected size (bp)							
		+ control	WT	Δ*bb0259*	*bb0259*[com]	+control	WT	E580Q	D606N
P1F	GACGACGGATCCCCCGAGCTTCAAGGAAGATTTCC								
P1R	GTGGTGAAGCTTTTAGAAAAACTCATCGAGCATC	1239	NA: No product	1239	1239	NA	NA	NA	NA
P2F	GTCGACGGATCCGTTATTTCTAATTCTGTTTTGTC								
P2R	CTGCAGTTAATTTTTGGGGAATTCGCCC	972	972	NA	972	NA	NA	NA	NA
P3F	CCTAACGTAAGCGGAGAATACAAGAGTCTTTTGCATTCTG								
P3R	CCCAAGCCTTGCATCAGCCCCATAAAAATTCCTGCTAAC	NA	NA	NA	NA	2292	1372	2292	2292

Figure S2. Confirmation of inactivation, complementation, and *bb0259* point mutants by PCR analysis. PCR products of *bb0259*-E580Q and -D606N were further sequenced to verify the mutations. Primer information and expected size of PCR products are shown in the table below the figure.

Figure S3

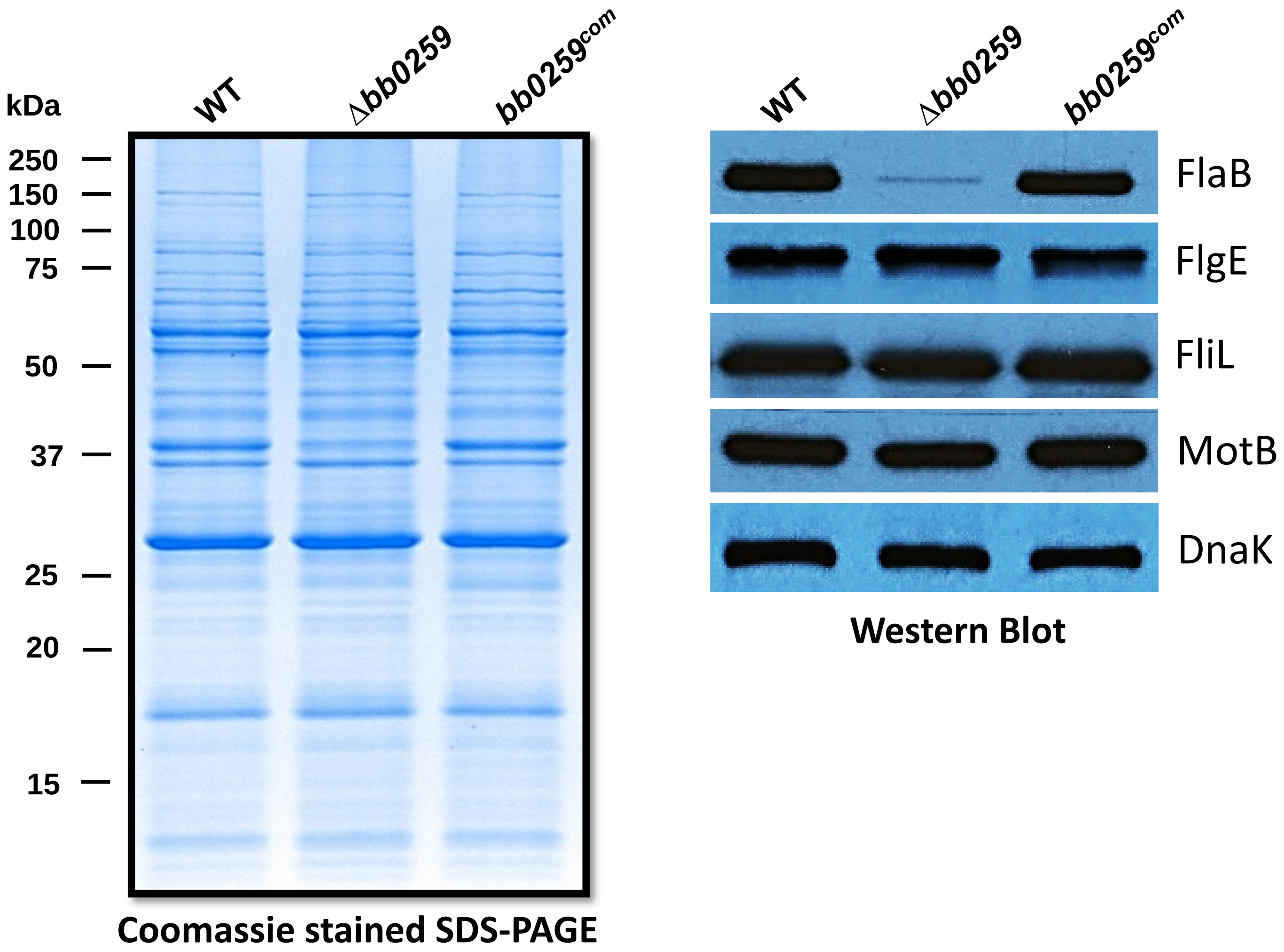

Figure S3. Effect of *bb0259* or *LTase*^{Bb} mutation on the synthesis of other flagellar proteins. Cell lysates were subjected to SDS-PAGE followed by Coomassie staining (left) or transferred to a PVDF membrane for immunoblot analysis (right). DnaK was used as a loading control.

Figure S4

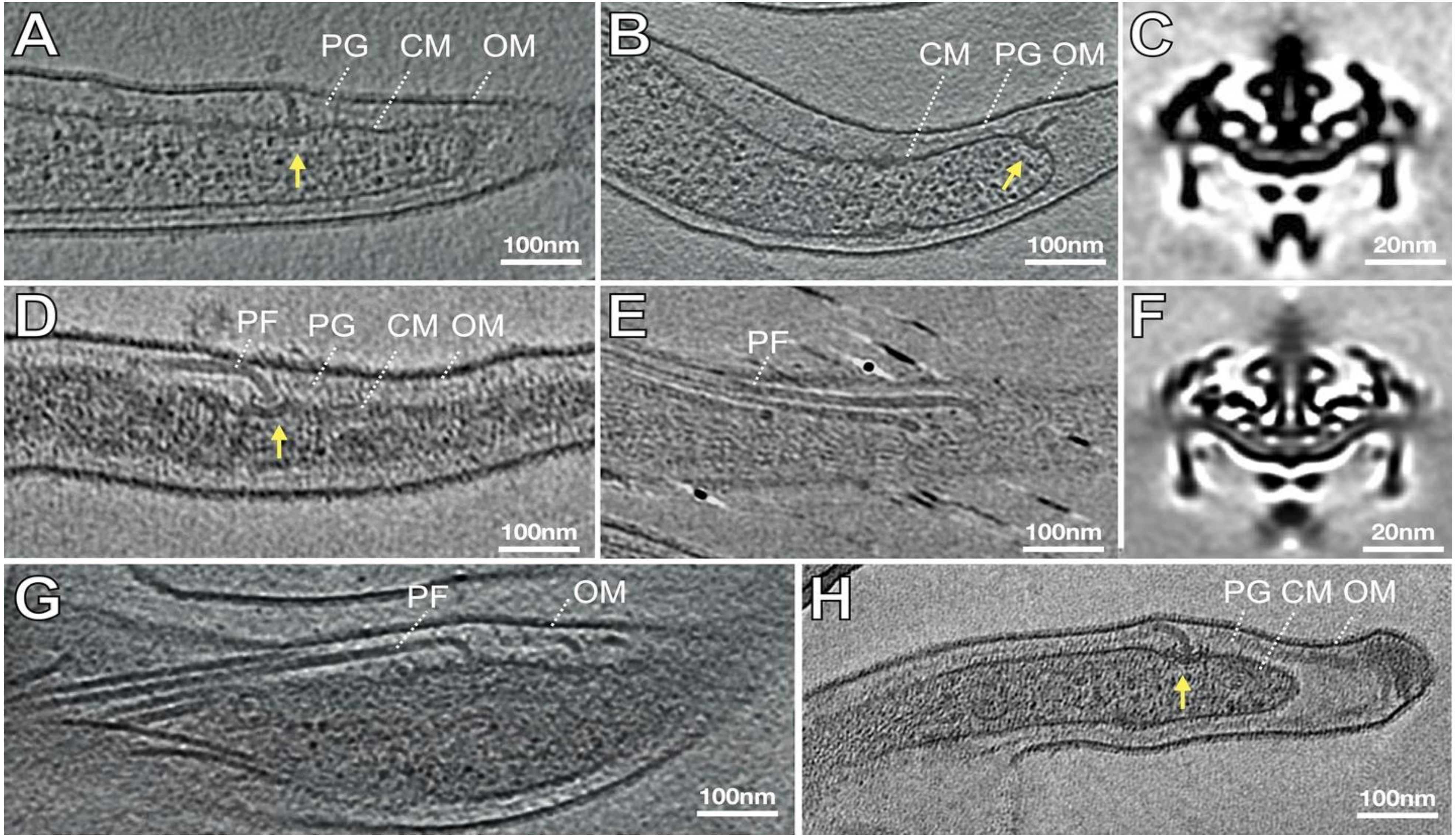

Figure S4. Cryo-ET analysis of Δ*bb0259* or *ΔLTase*^{Bb} and complemented *bb0259* strain. (A, B) Two representative tomographic slices from two Δ*bb0259* or *ΔLTase*^{Bb} cells show flagellar motors (arrows) and hooks. No filament is visible in the *Δbb0259* cells. (C) The averaged structure of the motor from the Δ*bb0259* cells. (D, E) Two representative tomographic slices from two WT cells show flagellar motors, hooks, and filaments. (F) The averaged structure of the motor from the WT cells. (G) A representative tomographic slice from a complemented cell shows periplasmic flagella (PF) with regular filaments. (H) A representative tomographic slice from a *flaB* mutant cell shows a motor with a hook. The hook penetrated the PG layer (See also Figure 5A of Zhao et al, 2013). CM, OM- cytoplasmic & outer membranes.

Figure S5

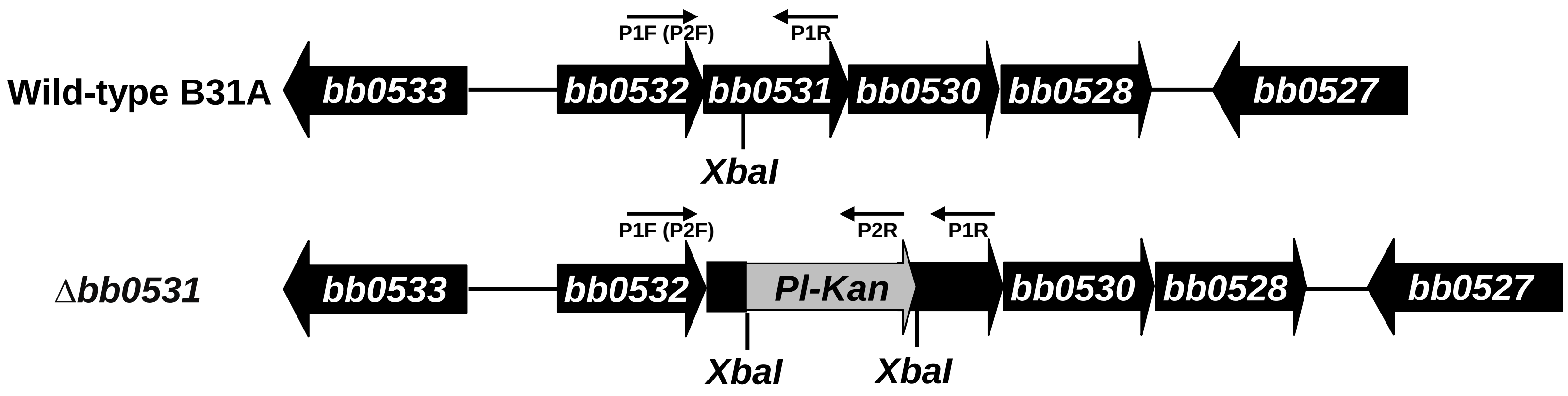

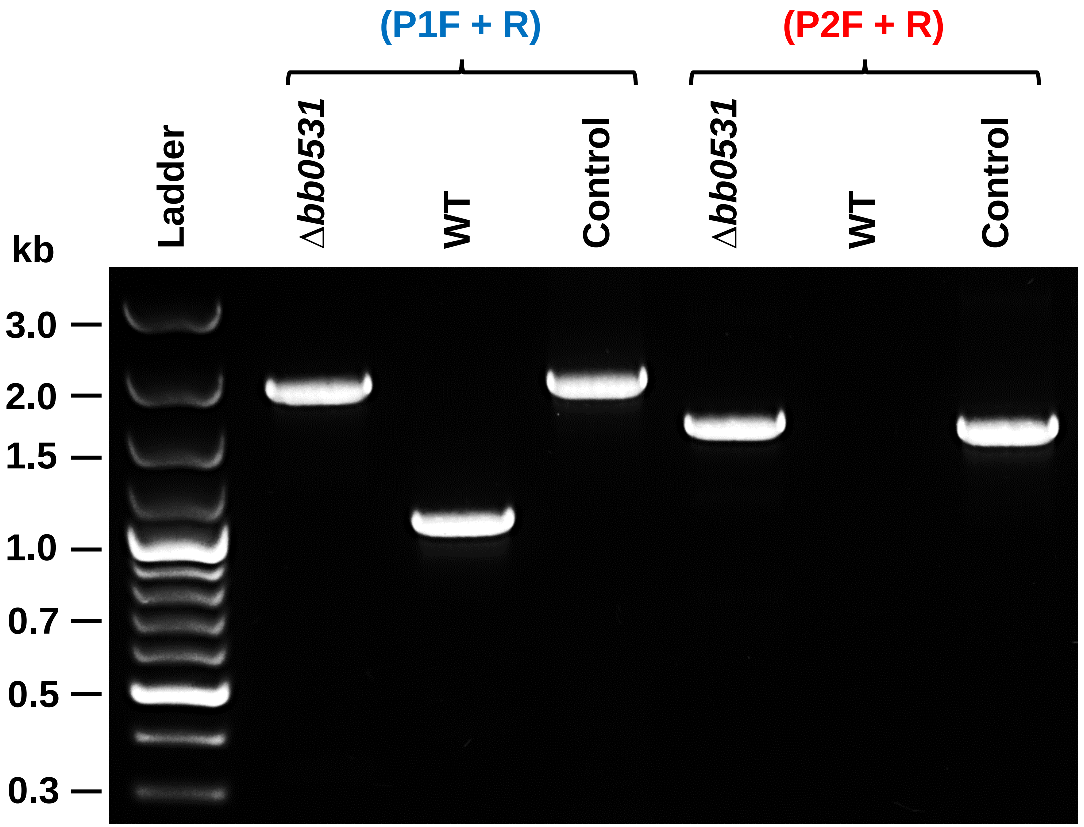

Primer name	Primer sequence	Expected size (bp)		
		+ control	WT	Δ*bb0531*
P1F	GCATTAGCTATTTAATTGAGCCTGCCAATATCC	1904	1052	1904
P1R	GTCGTAAAGTTTGTCTTTTGCCCATTTCCCAG			
P2F	GCATTAGCTATTTAATTGAGCCTGCCAATATCC	1531	NA	1531
P2R	gatgctcgatgagtttttctaaTCTAGAAATTAATAG			

Figure S5. Inactivation of *bb0531* and confirmation of the mutant by PCR. *bb0531* is located within a putative operon consisting of four genes (diagram is not to scale). The diagrams illustrate the construction of the mutant strain using a *Pl-Kan* cassette (Top panel). The mutant was confirmed by PCR and further verified by DNA sequencing. The primers used to confirm the mutant by PCR are shown in bottom right panel. The primer binding sites are shown in top panel.

Figure S6

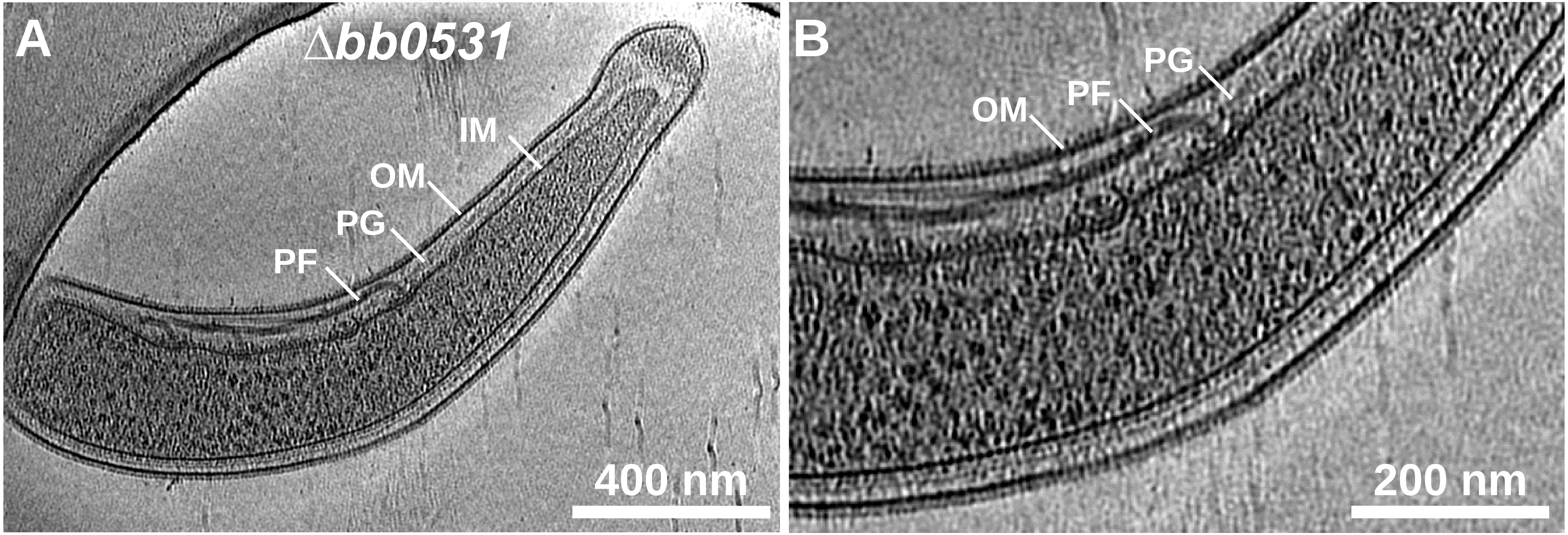

Figure S6. Cryo-ET reconstruction of a cell tip from a representative Δ*bb0531* mutant cell. (A) A slice from a cryo-ET reconstruction shows periplasmic flagellum (PF), outer membrane (OM), inner membrane (IM), and the peptidoglycan (PG) layer. (B) A zoom-in view from panel A.
